# Animal-assisted therapy on psychological and physical outcomes: a meta-analysis of randomised controlled trials

**DOI:** 10.7189/jogh.16.04236

**Published:** 2026-07-17

**Authors:** Chun-Ying Shih, Hidayat Arifin, Christina Yeni Kustanti, Wen-Jui Chang, Chi-Hsien Huang, Rizki Fitryasari, Mei-Chu Tsai, Jia-You Ye

**Affiliations:** 1 Department of Nursing, Chang Gung University of Science and Technology, Taoyuan, Taiwan; 2 Department of Medical-Surgical, Emergency, Disaster, and Critical Nursing, Faculty of Nursing, Universitas Airlangga, Surabaya, Indonesia; 3 Research Group in Medical-Surgical Nursing, Faculty of Nursing, Universitas Airlangga, Surabaya, Indonesia; 4 Study Program of Nursing Science, Sekolah Tinggi Ilmu Kesehatan Bethesda Yakkum, Yogyakarta, Indonesia; 5 Department of Thoracic Medicine, Chang Gung Memorial Hospital, Tucheng (New Taipei Municipal Tucheng Hospital) Branch, New Taipei City, Taiwan; 6 Department of Thoracic Medicine, Linkou Chang Gung Memorial Hospital, Taoyuan, Taiwan; 7 Department of Psychiatric/Mental Health Nursing, Faculty of Nursing, Universitas Airlangga, Surabaya, Indonesia; 8 Department of Nursing, Taoyuan Chang Gung Memorial Hospital, Taoyuan, Taiwan; 9 Post-Baccalaureate Program in Nursing, College of Nursing, Taipei Medical University, Taipei City, Taiwan

**Keywords:** animal-assisted therapy, meta-analysis, randomised controlled trials, psychological outcomes, adults, well-being

## Abstract

**Background:**

Adults are at heightened risk of anxiety, stress, and depression; animal-assisted therapy (AAT) may serve as an effective approach to promote psychological well-being. In this study, we compared the effectiveness of AAT in improving depression, anxiety, stress, pain, and gait among adults with or without illness.

**Methods:**

We systematically searched six electronic databases (PubMed, CINAHL, Embase, Web of Science, the Cochrane Library, and Scopus) and included all studies published up to August 2024. We used comprehensive meta-analysis software to complete the quantitative synthesis. We reported pooled effect sizes as Hedges’ g with corresponding 95% confidence intervals (CIs), after applying a random-effects model. Furthermore, we assessed heterogeneity using Cochran’s Q test and the *I*^2^ statistic. We applied the Cochrane Risk of Bias 2.0 tool to appraise the methodological quality of the included studies. The synthesis process followed PRISMA guidelines.

**Results:**

From the 13,345 studies identified, 35 randomised controlled trials involving 2391 adults were included. Across diverse populations, AAT was associated with reductions in depression (Hedges’ g = –0.403; 95% CI = –0.536, –0.271), anxiety (Hedges’ g = –0.661; 95% CI = –1.069, –0.253), and stress (Hedges’ g = –1.062; 95% CI = –1.849, –0.275) at post-intervention, although substantial between-study variability was observed.

**Conclusions:**

We demonstrated that AAT significantly improves anxiety, depression and stress in adults, but has no meaningful effect on pain or gait. Subgroup and meta-regression analyses indicate that psychological benefits depend on population and intervention characteristics, and are not moderated by age or gender.

**Registration:**

PROSPERO: CRD42024570108.

In the general adult population, the prevalence of anxiety was estimated to be 6.19%, stress to be 35.1%, and depression to be 12.9% [1–3]. Globally, the prevalence of such mental health disorders, including anxiety, stress, and depression, has shown steady increases, underscoring their growing significance as a public health concern, as evidenced by data from the World Health Organization (WHO) [4]. This phenomenon may be attributed to the rapid transformations in modern society, while individuals’ emotional adaptations have not fully kept pace, leading to increasingly significant psychological consequences over time [5,6]. By harnessing companionship *via* trained animals to foster deeper social connections, we can enhance psychological resilience and mitigate the risks of anxiety, stress, and depression, because interpersonal interactions are foundational to mental well-being, and within the framework of animal-assisted therapy (AAT), companionship serves as an essential and active therapeutic mechanism [7–9]. If left unaddressed, such psychological disturbances may further contribute to cognitive impairment or elevated mortality risks [10,11].

Non-pharmacological interventions are widely implemented to improve psychological outcomes, including anxiety, stress, and depression. In clinical settings, healthcare professionals play a pivotal role in administering these interventions owing to their continuous patient engagement, therapeutic communication competencies, and holistic approach to care. Previous nurse-led RCTs investigated a range of strategies, such as auditory relaxation using binaural beats [12], psychoeducation [13], behavioural activation programmes, and enhanced supportive care [14]. However, most interventions primarily targeted symptom reduction, with limited attention to the emotional and relational aspects of distress. As fostering comfort, connection, and emotional support is fundamental to nursing practice, companionship-based approaches warrant exploration. In this context, AAT may offer an innovative and practical strategy that nurses can integrate into holistic care to alleviate psychological distress and promote their patients’ emotional well-being [15,16].

AAT is a structured, goal-directed approach in which trained animals engage in planned interactions designed to support emotional and physical health. Commonly used therapy animals are dogs and horses, with robotic companions occasionally adopted as alternatives [15]. AAT has been applied across diverse settings such as hospitals and schools, benefiting both healthy individuals and those with medical conditions [17–20].

Current systematic reviews show that AAT is used across a broad range of populations and clinical conditions. Its applications extend to neurodegenerative disorders, for example, in individuals with Alzheimer disease [21]. and dementia [22–24], developmental disorders (*e.g.* autism spectrum disorder and attention-deficit/hyperactivity disorder) [19,25–27], psychiatric disorders (*e.g.* eating disorders, posttraumatic stress disorder, anxiety, and schizophrenia) [28–31], neurological conditions [32,33] (*e.g.* multiple sclerosis and cerebral palsy) [34,35], older adults [36,37], and critically ill patients [20].

Two prior systematic reviews are similar to the present study. The first examined AAT in adult populations but did not conduct a meta-analysis. Most of the studies were observational in design, and the population included those with dementia, depression, and posttraumatic stress disorder, which significantly influenced outcomes [27]. The second review focused on RCTs, the outcomes were rehabilitation effects, and its scope was limited to clinical populations [38]. Moreover, neither review analysed the effects of different animal species or intervention frequencies. In contrast, the present review targeted the general adult population and included only RCTs, providing a more rigorous and comprehensive evaluation of AAT effectiveness while also examining variations across animal types and intervention frequencies.

## METHODS

We conducted a meta-analysis to evaluate whether AAT improves psychological and physical outcomes in adults, encompassing both healthy individuals and those with medical conditions. We prepared the report in accordance with the PRISMA guidelines [39].

### Search strategy

We conducted a comprehensive search of Embase, Cochrane Library, CINAHL, PubMed, Web of Science, and Scopus (from inception to August 2024). We used the MeSH, Emtree, and free-text terms with combinations of keywords and Boolean operators, including animals (*e.g.* dog, cat, pet, animal) combined with animal-assisted therapy terms (*e.g.* assisted therapy, therapy, intervention). We identified additional relevant studies through a review of Google Scholar and other pertinent sources (Table S1 in the **Online Supplementary Document**).

### Eligibility criteria, study selection, and data extraction

We included only RCTs that satisfied the following criteria: adult participants (≥18 years) irrespective of health status; interventions involving animal-assisted therapy or animal-related interventions, defined in this review as structured interventions that intentionally incorporated direct or indirect interaction with live animals, animal-like robotic agents, or animal-related environmental exposure as part of a planned therapeutic or supportive intervention; a comparison condition of usual care (active or passive); and prespecified outcomes, with primary endpoints in psychological outcomes (depression, anxiety, and stress) and secondary endpoints in physical outcomes (pain and gait) (Table S2 in the **Online Supplementary Document**). We excluded studies if they were outside the target population or scope of this review, or if they were abstract-only, unpublished, ongoing trials, or study protocols. Extracted items comprised study identifiers (authors, year), participant characteristics (sample size, mean age, sex, setting, country, health status), and intervention/comparator details (AAT modality, control condition, frequency, session duration, total number of sessions, and delivery format). Outcome information included assessment time points and follow-up intervals. Psychological outcomes (depression, anxiety, stress) were prespecified as primary, with physical outcomes (pain, gait) designated as secondary. Disagreements were adjudicated by a third reviewer.

### Risk of bias assessment

Two reviewers independently performed data extraction. For assessing the risk of bias for each randomised trial, we used the RoB tool, version 2.0 (Cochrane, London, UK), which addresses five domains: the randomisation process, deviations from intended interventions, missing outcome data, outcome assessment, and the selection of results for reporting. In accordance with the guidance provided by Higgins *et al.*, each domain was categorised as ‘low risk of bias,’ ‘some concerns,’ or ‘high risk of bias’ [40]. We assessed publication bias by visually inspecting funnel plot symmetry and applying Egger’s regression to detect small-study effects, following the approach described by Egger *et al.* [41].

### Data analysis

We estimated pooled effects in Comprehensive Meta-Analysis, version 3.0 (Biostat, Englewood, New Jersey, USA). Effects were expressed as Hedges’ g with 95% CIs under a random-effects model; the effect sizes of 0.20–0.49, 0.50–0.79, and ≥0.80 denoted small, moderate, and large effects, respectively [42]. When multiple measures within the same outcome domain were reported, we prioritised validated instruments that directly assessed the target construct for data extraction. We used standardised mean differences (Hedges’ g) to synthesise conceptually comparable outcomes assessed using different instruments. Heterogeneity among studies was examined using Cochran’s Q together with the *I*^2^ statistic. Following the guidance of Higgins *et al.* [43], we considered *I*^2^ values of approximately 25%, 50%, and 75% to represent low, moderate, and high levels of heterogeneity. Subgroups examined categorical moderators: health status (healthy, mentally ill, physically ill), animal type (dog *vs.* other animals), animal format (real *vs.* robotic), intervention frequency (1–2, 3–4, or 5–7 sessions/week), and session duration (<30, ≥30 to ≤60, >60 minutes/session). Meta-regressions tested continuous moderators: mean age, percentage female, and total number of sessions. We considered the moderation effects statistically significant at *P* < 0.05. To assess the robustness of the pooled estimates and determine whether any single study disproportionately influenced the findings, leave-one-out sensitivity analyses were conducted for each outcome by sequentially excluding one study and recalculating the pooled effect size. In addition, sensitivity analyses were performed by excluding studies assessed as having a high risk of bias to examine whether the pooled estimates remained robust when restricted to studies at lower risk of bias [44].

## RESULTS

### Study selection

**Figure 1:**
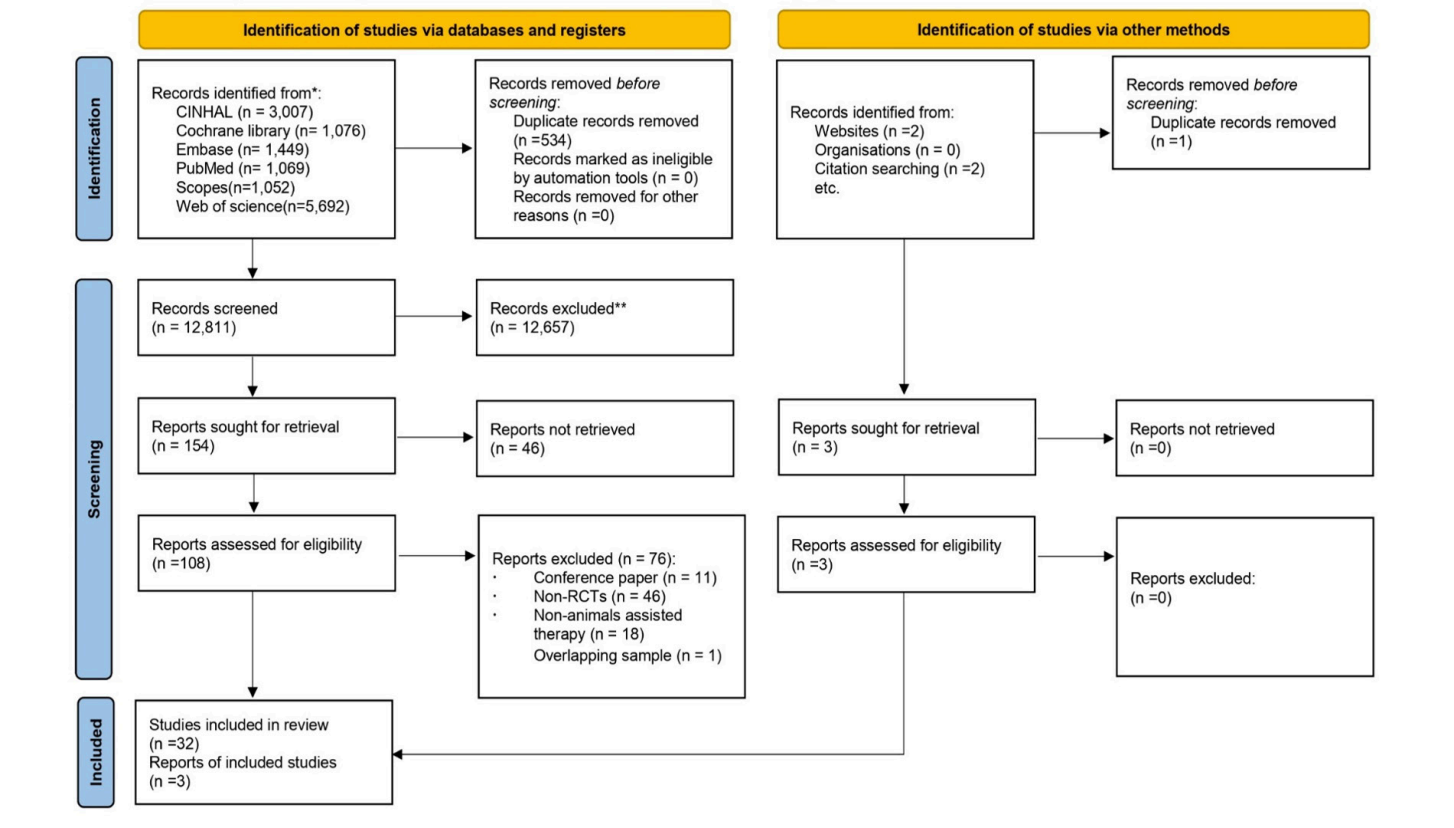
Preferred Reporting Items for Systematic Reviews and Meta-analyses flow diagram.

Through the database search, we identified 13,345 records across six electronic databases. After removing 534 duplicates, 12,811 records were screened by title and abstract, and 12,657 were excluded. Of the 154 reports sought for retrieval, 46 could not be obtained despite attempts to contact the corresponding authors. The remaining 108 reports were assessed for eligibility; 76 were excluded: conference papers (n = 11), non-RCTs (n = 46), studies not involving animal-assisted therapy (n = 18), and overlapping samples (n = 1). We identified four additional records through Google Scholar and citation searching; after removing one duplicate, three reports were assessed and included. Overall, we retained 35 RCTs for the final analysis (**Figure 1**).

### Characteristics of included studies

Of the included studies, 10 were conducted in the USA (28.6%), and 4 each in Italy and Norway (22.9%). The remaining 48.5% were conducted in Australia, Canada, France, Germany, Hungary, the Netherlands, New Zealand, South Africa, South Korea, Spain, Switzerland, and Taiwan. In total, 2391 adults, regardless of their health status, participated in the included studies. Information on gender was available for 31 studies, and in these studies, women formed the majority of the sample, with 1540 female participants out of 2190 individuals. Participant ages ranged from 18.9–85 years. The frequency of the interventions varied from one to seven sessions per week, and in a few protocols, the sessions were scheduled every two weeks or delivered at an intensive level of at least eight sessions per week. Session durations ranged 10–180 minutes per session. Among the 35 included studies, animal species varied, including dogs, birds, fish, dolphins, and other small or farm animals. Dogs were used in 71.4% of interventions. Regarding animal format, real animals accounted for 88.6% of interventions, while robotic animals accounted for 11.4% (Table S2 in the **Online Supplementary Document**).

### Risk of bias assessment

In the synthesis, 80% of the included studies were rated as having some concerns regarding the risk of bias according to the RoB 2.0 assessment. The randomisation process was generally appraised as having a high risk of bias. In contrast, the domains concerning deviations from the intended interventions, missing outcome data, and selection of reported results were most often rated as having some concerns or as being at low risk of bias. These findings indicated that although several RoB 2.0 domains were not consistently judged as high risk, the overall certainty of the evidence should be interpreted with caution because methodological concerns related to the randomisation process may have introduced important bias in the trial estimates (Table S3 in the **Online Supplementary Document**).

### Effects on primary outcomes: psychological variables

#### Depression

**Figure 2:**
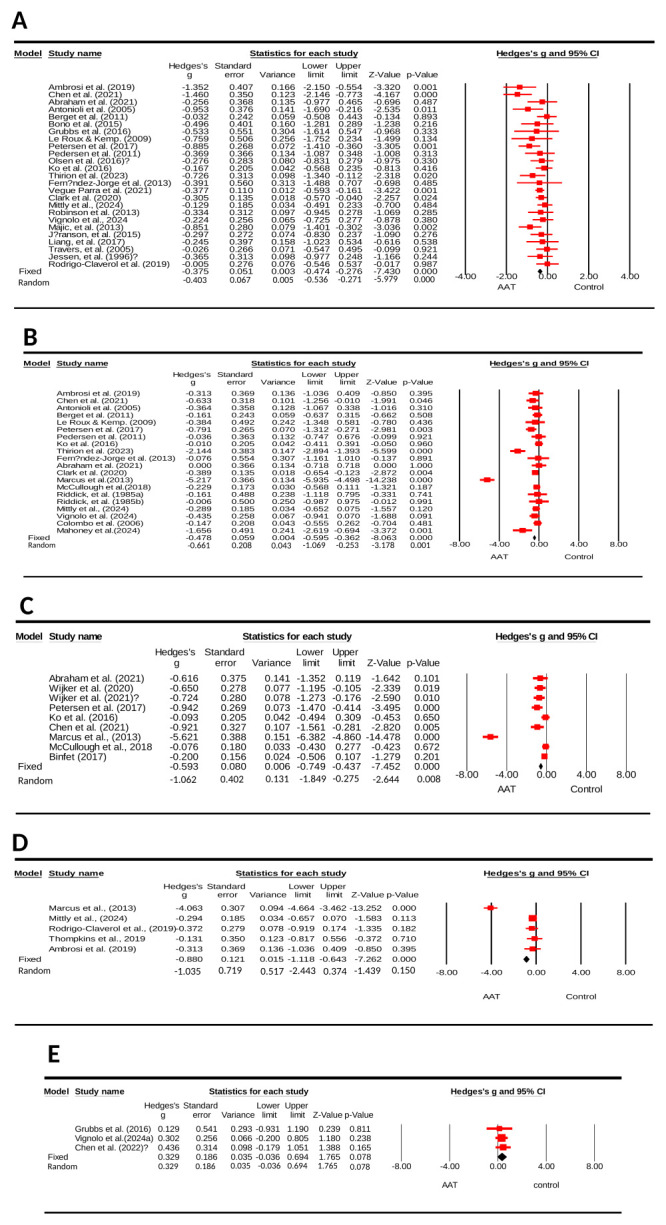
Forest plot comparing the AAT and control groups. **Panel A.** Depression. **Panel B.** Anxiety. **Panel C.** Stress. **Panel D.** Pain. **Panel E.** Gait.

We identified that 25 studies involving 1582 adult populations with different health conditions applied the Geriatric Depression Scale, including the 15-item version, short form, and short version (n = 8), the Cornell Scale for Depression in Dementia (n = 6), and the Beck Depression Inventory, including the second edition and the nine-item version (n = 5). Other measures included the Depression Anxiety Stress Scales-21 items (n = 1), the Patient Health Questionnaire-9 items (n = 1), the Hamilton Depression Rating Scale (n = 1), the Visual Analogue Scale for Depression (n = 1), the Hospital Anxiety and Depression Scale (n = 1), and the Depression in Mentally Aged Scale (n = 1) to assess the effect of AAT on depression. The pooled Hedges’ g for depressive symptoms was –0.403 (95% confidence interval (CI) = –0.536, –0.271), indicating a small to moderate effect in favour of AAT over the control conditions (*P* < 0.001). Low to moderate heterogeneity was observed across the studies included in the analysis (estimated τ^2^ = 0.032, Cochran’s Q (Q) = 35.59, *P* = 0.060, degrees of freedom (df) = 24, *I*^2^ = 32.58) (**Figure 2**, Panel A, **Table 1**). No evidence of publication bias was observed, and Egger’s regression test (*P* = 0.09). In the subgroup analyses, the type of population, type of animal, delivery format of the animal, session frequency, and per-session duration had significant effects on AAT on depression. Larger effects were observed in studies enrolling healthy participants, those using dogs, and those employing a robotic format. Greater reductions in depressive symptoms were also associated with delivering AAT with three to four sessions a week and with sessions lasting more than 60 minutes. By contrast, the meta-regression identified no covariates that were significantly associated with AAT’s antidepressant effect (**Table 2**; Table S4 in the **Online Supplementary Document**).

**Table 1:** Overall effect of AAT (random-effects model)

		Effect size				Heterogeneity			
**Outcome measurement**	**n**	**Pooled estimate (95% CI)**	**PI**	**Z-value**	***P*-value**	**Q-statistic**	***P*-value**	***I* ^2^**	**τ^2^**
**Primary outcomes**									
Psychological									
*Depression*	25	–0.403 (–0.536, –0.271)	–0.7988, –0.002	–5.979	<0.001	35.59	0.060	32.58	0.032
*Anxiety*	20	–0.661 (–1.069, –0.253)	–2.5276, 1.2056	–3.178	0.001	211.98	<0.001	91.04	0.746
*Stress*	9	–1.062 (–1.849, –0.275)	–4.4394, 1.2354	–2.644	0.008	197.00	<0.001	95.72	1.424
**Secondary outcomes**									
Physical									
*Pain*	5	–1.035 (–2.443, 0.374)	–6.5536, 4.4836	–1.439	0.150	128.02	<0.001	96.88	2.491
*Gait*	3	0.329 (–0.036, 0.694)	–2.0372, 2.6952	1.765	0.078	0.263	0.877	0.00	0

CI – confidence interval, PI – prediction interval

**Table 2:** Moderators of the subgroup analysis for depression and anxiety in adults

	Depression (n = 25)			Anxiety (n = 20)		
**Variable**	**n**	**Coefficient**	***P-*value**	**n**	**Coefficient**	***P-*value**
Type of population			<0.001			0.002
*Healthy*	5	–0.548		7	–0.286	
*Mentally ill*	16	–0.471		8	–0.532	
*Physically ill*	4	–0.213		5	–1.267	
Type of animal			<0.001			0.004
*Dog*	17	–0.421		12	–0.970	
*Others (including birds, horses, and fish, etc.)*	8	–0.374		8	–0.210	
Format of animal			<0.001			<0.001
*Real*	21	–0.395		19	–0.655	
*Robotic*	4	–0.480		1	–0.791	
Frequency (sessions per week)			<0.001			0.001
*Low (1–2)*	16	–0.419		13	–0.906	
*Moderate (3–4)*	2	–0.818		2	–0.448	
*High (5–7)*	2	–0.622		1	–0.364	
Duration in minutes			<0.001			0.001
*Short (<30)*	2	–0.549		7	–1.204	
*Medium (≥30 to ≤60)*	16	–0.375		7	–0.544	
*Long (>60)*	5	–0.603		3	–0.321	

CI – confidence interval

#### Anxiety

The present analysis analysed 20 studies involving 1253 adults across diverse populations. Anxiety outcomes were primarily assessed using the State-Trait Anxiety Inventory (including subscale uses; n = 9), followed by the Beck Anxiety Inventory (n = 2). Other measures included the Generalised Anxiety Disorder-7 (n = 1), Depression Anxiety Stress Scales-21 (n = 1), Zung Self-Rating Anxiety Scale (n = 1), Rating Anxiety in Dementia (n = 1), Hospital Anxiety and Depression Scale (n = 1), the Brief Symptom Inventory (n = 1), Numeric Rating Scale (n = 1), Patient Health Questionnaire-15 (n = 1), and Visual Analogue Scale (n = 1).

The pooled Hedges’ g was –0.661 (95% CI = –1.069, –0.253), indicating a statistically significant anxiety reduction associated with AAT (*P* = 0.001). However, heterogeneity was substantial (τ^2^ = 0.746, Q = 211.98, *P* < 0.001, *I*^2^ = 91.04), suggesting considerable variability across studies; therefore, this pooled estimate should be interpreted with caution (**Figure 2**, Panel B, **Table 1**). No evidence of publication bias was observed, based on visual inspection of the funnel plot and a non-significant Egger’s regression test (*P* = 0.24). Subgroup analyses suggested that population characteristics, type of animal, delivery format, session frequency, and session duration may be associated with variability in effect sizes. Larger effects were observed in studies enrolling physically ill participants, those using dogs, and those employing a robotic format. Greater reductions in anxiety were also associated with delivering AAT through one to two sessions/week and with sessions lasting less than 30 minutes. However, meta-regression analyses did not identify any statistically significant moderators (**Table 2**; Table S4 in the **Online Supplementary Document**).

#### Stress

In the analysis of stress outcomes, nine studies involving 750 adults across diverse populations were included. The Perceived Stress Scale was the most commonly used instrument (n = 4). Other measures included Galvanic Skin Response (n = 1), the Brief Encounter Psychosocial Instrument (n = 1), the Depression Anxiety Stress Scales–21 items (n = 1), the Coping Scale (n = 1), and the Parenting Stress Index–Parent Impact Questionnaire (n = 1). The pooled Hedges’ g was –1.062 (95% CI = −1.849, −0.275), indicating a statistically significant reduction in stress associated with AAT (*P* = 0.008). However, heterogeneity was substantial (τ^2^ = 1.424, Q = 197.00, *P* < 0.001, *I*^2^ = 95.72), suggesting considerable variability across studies. Therefore, this pooled estimate should be interpreted with caution (**Figure 2**, Panel C, **Table 1**). Evidence of potential publication bias was indicated by Egger’s regression test (*P* = 0.05). However, trim-and-fill analysis showed that the adjusted pooled effect size was similar to the original estimate, suggesting that the overall findings were relatively robust. Subgroup analyses suggested exploratory patterns broadly consistent with those observed for anxiety. Population characteristics, animal type, delivery format, session frequency, and session duration appeared to be associated with variability in effect sizes. Larger effects were observed in studies involving physically ill participants, interventions delivered one to two sessions per week, and sessions lasting less than 30 minutes. Studies using real animals also appeared to show larger effects than those using robotic formats. However, given the limited number of studies on the stress outcome, these subgroup findings should be interpreted with caution. Meta-regression analyses did not identify any statistically significant moderators (Tables S4 and S5 in the **Online Supplementary Document**).

### Effects on secondary outcomes: physical function

#### Pain

In the analysis, five of the studies included pain, encompassing 361 adult populations with different health conditions, utilising a visual analogue scale (n = 2), a numeric rating scale (n = 2), and the Brief Pain Inventory (n = 1) to examine the effect of AAT on each individual’s pain. The findings revealed a large pooled Hedges’ g effect size estimate of –1.035 (95% CI = –2.443, 0.374), indicating no significant difference between the AAT and control groups in pain (*P* = 0.150). A high level of heterogeneity was noted among the five studies (estimated τ^2^ = 2.491, Q = 128.02, *P* < 0.001, df = 4, *I*^2^ = 96.88) (**Figure 2**, Panel D, **Table 1**). Egger’s regression test indicated no meaningful evidence of publication bias (*P* = 0.67).

#### Gait

Regarding gait, three studies examined gait in 112 adult populations with different health conditions and utilised the 6-Minute Walk Test (n = 2) and the 5-Meter Walk Test (n = 1) to assess the effect of AAT on each individual’s gait performance. No statistically significant difference in gait performance was found between the AAT and control groups, with a pooled Hedges’ g of 0.329 (95% CI = –0.036, 0.694, *P* = 0.078). The three studies demonstrated a low level of heterogeneity (estimated τ^2^ = 0.00, Q = 0.263, *P* = 0.877, df = 2, *I*^2^ = 0) (**Figure 2**, Panel E, **Table 1**). No indication of publication bias was detected by Egger’s regression test, which yielded *P* = 0.64.

#### Sensitivity analysis of all outcome variables

For depression and anxiety, the direction and statistical significance of the pooled effects remained consistent after sequential exclusion of individual studies. The recalculated pooled effects ranged from −0.358 to −0.424 for depression and from −0.394 to −0.700 for anxiety, suggesting that these findings were not driven by any single study. Similar patterns were observed for stress, although the range of recalculated pooled effects was wider, ranging from −0.461 to −1.195. In contrast, the pain outcome showed greater variability in the recalculated pooled effects, ranging from −0.291 to −1.258, indicating relatively lower robustness. For gait, the pooled effect remained statistically non-significant after sequential exclusion of individual studies, with recalculated pooled effects ranging from 0.271 to 0.359. Sensitivity analyses excluding studies at high risk of bias were further conducted across outcomes (Tables S5–7 in the **Online Supplementary Document**). The pooled effects for depression, anxiety, and stress remained statistically significant after exclusion of high-risk studies, suggesting that these findings were generally robust. In contrast, the pooled effect for pain remained non-significant after excluding high-risk studies, indicating lower robustness. No high-risk-of-bias studies contributed data to the gait outcome.

## DISCUSSION

The present meta-analysis, which synthesised 35 RCTs with a total of 2391 participants, provides a broad synthesis of randomised evidence on the potential effects of AAT in improving psychological (*i.e.* anxiety, depression, and stress) and physical outcomes (*i.e.* pain and gait) among adults. While several previous reviews examined the effects of AAT in specific clinical populations, few comprehensively evaluated its overall impact across the adult populations with different health conditions. Previous systematic reviews identified only three RCTs in adult populations, among which two reported significant reductions in depressive symptoms compared to control groups, and one found no significant effect [27]. Results of the current meta-analysis suggested statistically significant reductions in anxiety, depression, and stress, although these findings should be interpreted cautiously because of substantial heterogeneity. No significant effects were observed for physical outcomes such as pain and gait. Moreover, the high degree of heterogeneity observed across several outcomes limits the certainty, interpretability, and generalisability of these pooled estimates. These results may vary according to differences in participant populations, animal types or species, intervention formats, session frequency or duration, and outcome measures. Future studies should further investigate these potential sources of heterogeneity to better clarify the mechanisms underlying the effects of AAT.

### Effects of AAT on primary outcomes: anxiety, depression, and stress

The findings of this study suggested statistically significant reductions in psychological outcomes, including anxiety, depression, and stress, compared to control groups. One possible explanation for these observed effects is the companionship provided during AAT interventions, whether involving real or mechanical animals, which may offer emotional support and interactive experiences that support psychological well-being [15,16]. These findings may be consistent with the proposed role of companionship in emotional regulation and stress reduction. Previous studies have similarly suggested that AAT may help reduce psychological distress across various populations [45–47]. However, variations in intervention parameters, such as frequency, duration, animal species, and the use of real *vs.* robotic animals, as well as differences in the assessment instruments used to evaluate psychological outcomes, may have collectively contributed to the inconsistencies observed across studies. For depression scales, the most commonly used instruments were the Depression Anxiety Stress Scales-21 and the Hospital Anxiety and Depression Scale. However, because these tools encompass different item structures and conceptual domains, the effect sizes derived from them may vary, leading to differences in the measured outcomes [48].

Future research should systematically investigate these factors and examine different intervention ‘dosages,’ such as variations in frequency, duration, and intensity, to clarify how these factors may influence the observed effects of AAT. In addition, network meta-analysis may be useful for exploring the comparative effects of different animal types and intervention characteristics, provided that sufficient and clinically comparable studies become available.

### Effects of AAT on secondary outcomes: pain and gait

Results of this study indicated that AAT did not significantly improve physiological outcomes such as pain and gait. However, these findings should be interpreted with caution because only a limited number of studies assessed these outcomes, yielding insufficient evidence. Consequently, subgroup analyses (*e.g.* examining differences by intervention dosage) and meta-regression analyses (*e.g.* exploring variations by age or gender) could not be performed for these outcomes [49,50]. AAT, however, primarily focuses on providing emotional support and companionship rather than directly modulating pain mechanisms, which may partly explain why clear effects on pain symptoms were not observed [15,16].

As for gait, the absence of statistically significant improvement may partly reflect the characteristics of the study populations, as most participants were older adults, who are more susceptible to muscle weakness and impaired coordination, thereby constraining the potential for functional enhancement [51,52]. Furthermore, the duration and frequency of AAT interventions may have been insufficient to induce measurable physiological adaptations.

From a mechanistic perspective, the association between AAT and physiological outcomes, such as pain and gait, remains relatively poorly understood. Future research should therefore include a greater number of studies focusing on these domains and explore whether varying intervention dosages, such as differences in frequency, duration, or intensity, influence the effects of AAT on pain and gait performance.

### Moderator analysis meta-regression in the present study

Subgroup analyses were performed to examine categorical moderators, including the population’s health status, type and format of animals, intervention frequency, and session duration, whereas meta-regression analyses explored continuous moderators such as mean age and gender distribution. Given the small number of studies in several subgroup categories and the substantial heterogeneity across outcomes, these findings should be interpreted as exploratory and hypothesis-generating. Considering the recurrent and chronic nature of depression [53], exploratory subgroup findings suggesting larger effects in studies with longer session durations and higher intervention frequencies may indicate that longer and more frequent interventions are more likely to show observable effects. In addition, studies using robotic animals appeared to show larger effects than those using real animals. Regarding animal format, the larger effects observed in studies using robotic animals may be attributed to their ability to simulate real animal behaviours and responsiveness, thereby providing a more consistent and comprehensive sense of companionship [24,54]. Anxiety symptoms may fluctuate in intensity and can be influenced by situational stressors or immediate emotional experiences [55]. Therefore, the exploratory subgroup findings showing larger effects in studies with shorter session durations and lower intervention frequencies may reflect the context-sensitive nature of anxiety symptoms, although this interpretation should be considered tentative.

Stress responses are often characterised by acute psychological and physiological reactions to stressors [55]. Consistent with this mechanism, exploratory subgroup findings suggested larger effects in studies with shorter session durations (<30 minutes) and lower intervention frequencies (one to two sessions per week). Studies involving real dogs appeared to show larger effects than those using robotic dogs. This may be because tactile interaction with live animals provides more immediate emotional or sensory engagement, although this interpretation remains speculative and should be approached with caution.

The present meta-regression analysis did not identify statistically significant gender-related moderation effects for anxiety, depression, or stress outcomes. However, these findings should be interpreted cautiously because the limited number of included studies may have reduced the statistical power of the meta-regression analyses. Therefore, the nonsignificant findings should not be interpreted as evidence of the absence of gender-related effects. Previous studies have suggested that women may be more susceptible to anxiety, depression, and stress [56]. In the present meta-analysis, only one study included participants younger than 20 years, whereas most of the remaining studies involved participants predominantly in middle-aged or older-adult ranges, with relatively few younger adults represented. Age-related differences in psychological outcomes may therefore warrant further investigation in future studies [57,58].

### Research implication

This meta-analysis suggests that AAT may have potential as a non-pharmacological adjunct to usual care for improving psychological outcomes in adults, including anxiety, depression, and stress, although the certainty of these findings is limited by substantial heterogeneity. No clear benefits were observed for physical outcomes, including pain and gait. Exploratory subgroup findings suggested that effect estimates may vary by participant health status, animal type, intervention format, session frequency, and session duration. However, because several subgroup categories included only a small number of studies, these patterns should not be interpreted as evidence of superior intervention components or optimal regimens. However, these subgroup findings should be interpreted as exploratory and hypothesis-generating rather than as evidence of optimal intervention regimens. Age and gender did not significantly moderate effects. Given the exploratory nature of these analyses, implementation decisions should also consider clinical indication, feasibility, safety, and participant preference. For translation into practice, AAT programmes should incorporate standardised screening and safety procedures (*e.g.* allergy risk, fear/avoidance, infection prevention, and animal welfare), clear intervention protocols with fidelity monitoring, and systematic documentation of adherence and adverse events to support consistent and safe integration across clinical and community settings.

### Strengths and limitations of this study

Our systematic review and meta-analysis employed a rigorous methodology, including only RCTs, thereby enhancing the comparability and internal validity of the results. However, several limitations should be acknowledged. First, the overall quality of the included studies inherently determined the quality of the synthesised evidence. Notably, according to our appraisal criteria, some of the included studies demonstrated suboptimal methodological quality. Several exhibited potential risks of bias, such as inadequate or unreported allocation concealment procedures and a lack of clarity regarding outcome assessments. Second, several studies included in this meta-analysis had relatively small sample sizes, which may have affected the precision and robustness of the pooled effect estimates, consequently limiting the internal validity of the findings. Third, although subgroup analyses were conducted to explore potential sources of heterogeneity, these findings should be interpreted with caution because multiple subgroup comparisons were performed and several subgroup categories included only a small number of studies. Therefore, the subgroup findings should be regarded as exploratory and hypothesis-generating rather than confirmatory. Finally, the use of diverse outcome measures and assessment tools across studies may have contributed to heterogeneity and affected the estimated magnitude of the pooled effect sizes.

## CONCLUSIONS

This meta-analysis demonstrated that AAT was associated with improvements in psychological outcomes, including anxiety, depression, and stress, but showed no significant effects on physical outcomes, including pain and gait, among adults. Subgroup analyses suggested that the delivery characteristics associated with larger effects varied by outcome. Depression showed greater effects in studies involving healthy participants, dog-assisted or robotic-format interventions, sessions lasting >60 minutes, and delivery three to four times per week. Anxiety showed greater effects in studies involving adults with physical illnesses, dog-assisted interventions, low-frequency delivery, and sessions lasting <30 minutes. Stress showed greater effects in studies involving adults with physical illnesses, real-dog interventions, one to two sessions per week, and sessions lasting <30 minutes. Meta-regression results further indicated that neither age nor gender significantly moderated psychological outcomes. Future studies with larger sample sizes and higher methodological quality are needed to validate these subgroup findings.

**Data availability:** The datasets used and/or analysed during the current study are available from the corresponding author on reasonable request.

## Additional material


Online Supplementary Document
