# Supplementary material for: Animal-assisted therapy on psychological and physical outcomes: a meta-analysis of randomised controlled trials
Source: J Glob Health. 2026 Jul 17;16:04236. doi: 10.7189/jogh.16.04236 (PMC13377760; doi:10.7189/jogh.16.04236)
Supplement: Online Supplementary Document [file jogh-16-04236-s001.pdf]

Supplement to: Shih CY, Hidayat A, Kustanti CY, Chang WJ, Huang CH, Fitryasari R, Tsai MC, Ye JY. Animal-assisted therapy on psychological and physical outcomes: a meta-analysis of randomised controlled trials. J Glob Health. 2026;16:04236.

Table S1A. Search term

Databases	Population	Intervention	Study design
PubMed	- Persons (MeSH)<br>- Adult (MeSH)	- Animal Assisted Therapy (MeSH)<br>- Animals (MeSH)<br>- Animals Population Group (MeSH)<br>- Therapy Animals (MeSH)<br>- Human-Animal Bond (MeSH)<br>- Human-Animal Interaction (MeSH)<br>- Canidae (MeSH)<br>- Felis (MeSH)<br>- Rodentia (MeSH)<br>- Birds (MeSH)<br>- Galliformes (MeSH)<br>- Animal Assisted Therapies<br>- Animal Facilitated Therapy<br>- Companion animal<br>- Pet Facilitated Therapy<br>- Pet Therapy<br>- Dogs<br>- Cats	- Randomized Controlled Trial (MeSH)
EMBASE	- Adult (Emtree)	- Animal assisted therapy (Emtree)<br>- Animal (Emtree)<br>- Human-animal relation (Emtree)	- Randomized controlled trial (Emtree)

Databases	Population	Intervention	Study design
Cochrane Library	- Persons (MeSH)<br>- Adult (MeSH)	- Therapy animal (Emtree)<br>- Canidae (Emtree)<br>- Rodent (Emtree)<br>- Bird (Emtree)<br>- Felidae (Emtree)<br>- Domestic animal (Emtree)<br>- Animal Facilitated Therapy<br>- Dogs<br>- Cats<br>- Animal Assisted Therapy (MeSH)<br>- Animals (MeSH)<br>- Animals Population Group (MeSH)<br>- Therapy Animals (MeSH)<br>- Human-Animal Bond (MeSH)<br>- Human-Animal Interaction (MeSH)<br>- Canidae (MeSH)<br>- Felis (MeSH)<br>- Rodentia (MeSH)<br>- Birds (MeSH)<br>- Galliformes (MeSH)<br>- Animal Assisted Therapies<br>- Animal Facilitated Therapy<br>- Companion animal<br>- Pet Facilitated Therapy<br>- Pet Therapy	- Randomized Controlled Trial (MeSH)

Databases	Population	Intervention	Study design
CINAHL	- Named Groups (CINAHL SH)<br>- Adult (CINAHL SH)	- Dogs<br>- Cats<br>- Pet Therapy (CINAHL SH)<br>- Animals (CINAHL SH)<br>- Animals Population Group (CINAHL SH)<br>- Therapy Animals (CINAHL SH)<br>- Human-Pet Bonding (CINAHL SH)<br>- Human-Animal Interaction (CINAHL SH)<br>- Dogs (CINAHL SH)<br>- Cats (CINAHL SH)<br>- Rodents (CINAHL SH)<br>- Birds (CINAHL SH)<br>- Canidae<br>- Felis<br>- Galliformes<br>- Animal Assisted Therapies<br>- Animal Facilitated Therapy<br>- Companion animal<br>- Pet Facilitated Therapy	- Randomized Controlled Trials (CINAHL SH)
Web of Science	- Persons (MeSH)<br>- Adult (MeSH)	- Animal Assisted Therapy (MeSH)<br>- Animals (MeSH)<br>- Animals Population Group (MeSH)<br>- Therapy Animals (MeSH)	- Randomized Controlled Trial (MeSH)

Databases	Population	Intervention	Study design
		- Human-Animal Bond (MeSH)<br>- Human-Animal Interaction (MeSH)<br>- Canidae (MeSH)<br>- Felis (MeSH)<br>- Rodentia (MeSH)<br>- Birds (MeSH)<br>- Galliformes (MeSH)<br>- Animal Assisted Therapies<br>- Animal Facilitated Therapy<br>- Companion animal<br>- Pet Facilitated Therapy<br>- Pet Therapy<br>- Dogs<br>- Cats	
Scopus	- Persons (MeSH)<br>- Adult (MeSH)	- Animal Assisted Therapy (MeSH)<br>- Animals (MeSH)<br>- Animals Population Group (MeSH)<br>- Therapy Animals (MeSH)<br>- Human-Animal Bond (MeSH)<br>- Human-Animal Interaction (MeSH)<br>- Canidae (MeSH)<br>- Felis (MeSH)<br>- Rodentia (MeSH)<br>- Birds (MeSH)<br>- Galliformes (MeSH)	- Randomized Controlled Trial (MeSH)

Databases	Population	Intervention	Study design
		- Animal Assisted Therapies<br>- Animal Facilitated Therapy<br>- Companion animal<br>- Pet Facilitated Therapy<br>- Pet Therapy<br>- Dogs<br>- Cats	

Table S1B. Syntax for the literature search of the databases.

Database	Keywords	Number of studies
CINAHL	CINAHL plus Sat, 8/26, 2024 12:39:23<br>S1 canidae OR felis OR rodentia OR birds OR galliformes OR dog OR cat OR animal OR pet OR Human-Animal 585,673<br>S2 assisted therapy OR population group OR therapy OR therapies OR bond OR interaction OR Assisted Therapies OR Facilitated Therapy OR Companion OR intervention OR treatment 3,327,394<br>S3 Randomized Controlled Trials 215,118<br>S4 S1 AND S2 AND S3 3,007	3,007
Cochrane Library	Search time:2024/08/26<br>canidae OR felis OR rodentia OR birds OR galliformes OR dog OR cat OR animal OR pet OR Human-Animal in Record Title AND (assisted therapy OR population group OR therapy OR therapies OR bond OR interaction OR Assisted Therapies OR Facilitated Therapy OR Companion OR intervention OR treatment in Record Title - (Word variations have been searched)	1,076
EMBASE	Search time:2024/08/26<br>(canidae:ab,ti OR felis:ab,ti OR rodentia:ab,ti OR birds:ab,ti OR galliformes:ab,ti OR dog:ab,ti OR cat:ab,ti OR animal:ab,ti OR pet:ab,ti OR 'human animal':ab,ti) AND ('assisted therapy':ab,ti OR 'population group':ab,ti OR therapy:ab,ti OR therapies:ab,ti OR bond:ab,ti OR interaction:ab,ti OR 'assisted therapies':ab,ti OR 'facilitated therapy':ab,ti OR companion:ab,ti OR intervention:ab,ti OR treatment:ab,ti) AND 'randomized controlled trial':ab,ti	1,449
PubMed	Search time:2024/08/26, 12:10:34<br>((((((((((canidae[Title/Abstract]) OR (felis[Title/Abstract])) OR (rodentia[Title/Abstract])) OR (birds[Title/Abstract])) OR (galliformes[Title/Abstract])) OR (dog[Title/Abstract])) OR (cat[Title/Abstract])) OR (animal[Title/Abstract])) OR (pet[Title/Abstract])) OR (Human-Animal[Title/Abstract])) AND (((((((((((assisted therapy[Title/Abstract]) OR (population group[Title/Abstract])) OR (therapy[Title/Abstract])) OR (therapies[Title/Abstract])) OR (bond[Title/Abstract])) OR (interaction[Title/Abstract])) OR (Assisted Therapies[Title/Abstract])) OR (Facilitated Therapy[Title/Abstract])) OR (Companion[Title/Abstract])) OR (intervention[Title/Abstract])) OR (treatment[Title/Abstract]))) AND (Randomized Controlled Trials[Title/Abstract])	1,069
Web of Science	# Web of Science Search Strategy (v0.1)<br><br># Database: Web of Science Core Collection<br><br># Entitlements:<br><br>- WOS.SCI: 1971 to 2024<br>- WOS.SSCI: 1971 to 2024	5,692

	# Searches:<br><br>1: AB=(canidae OR felis OR rodentia OR birds OR galliformes OR dog OR cat OR animal OR pet OR Human-Animal)<br>Date Run: Mon Aug 26 2024 00:30:54 GMT+0800 (台北標準時間) Results: 1858260<br><br>2: AB=(assisted therapy OR population group OR therapy OR therapies OR bond OR interaction OR Assisted Therapies OR Facilitated Therapy OR Companion OR intervention OR treatment)<br>Date Run: Mon Aug 26 2024 00:31:20 GMT+0800 (台北標準時間) Results: 10495455<br><br>3: AB=(Randomized Controlled Trial)<br>Date Run: Mon Aug 26 2024 00:31:38 GMT+0800 (台北標準時間) Results: 324809<br><br>4: #3 AND #2 AND #1<br>Date Run: Mon Aug 26 2024 00:31:48 GMT+0800 (台北標準時間) Results: 5692	
Scopus	Search time:2024/08/26, 12:09<br>TITLE-ABS-KEY (canidae OR felis OR rodentia OR birds OR galliformes OR dog OR cat OR animal OR pet OR human-animal) AND TITLE-ABS-KEY (assisted AND therapy OR population AND group OR therapy OR therapies OR bond OR interaction OR assisted AND therapies OR facilitated AND therapy OR companion OR intervention OR treatment) AND TITLE-ABS-KEY (randomized AND controlled AND trials)	1,052
Total		13,345

Table S2. Characteristics of included studies

Study	Demographic		Interventions			Quality appraisal
			Procedure	Outcomes/measurement tools	Evaluation/follow-up	RoB 2.0
Abraham et al. 2021 [1]	Sample size overall: 29, EG: 12 (41.4%), CG: 17 (58.6%). Mean (SD) age in years: EG: 31.0±7.0, CG: 33.0±8.0, Overall: 32.17±7.54. Gender: M: 21 (72.4%), F: 8 (27.6%)	Country:USA; Setting: community: 29 (100%) hospital: NA, institution:NA; Population: posttraumatic stress disorder	Experimental: Service dog training program. Frequency: 6 sessions in 2 weeks. Duration: 60 minutes. Total sessions: 6. Format: mixed animal: dog. Control: waiting list control	Physical: Sleep PIRSPsychological:PTSD symptoms PCL-CStressPSSDepressionPHQ-9MoodPOMSResilienceCDRISAnxietyPHQ-15Others: Social supportMOS-SSSSomatic symptomsPHQ-15	Baseline, Post-test (2 weeks)	Some concerns
Ambrosi et al. (2019) [2]	Sample size<br>Overall: 31<br>EG: 17 (54.8%)<br>CG: 14 (45.2%)<br>Mean (SD) age (years):<br>EG: 82.6±NI<br>CG: 87.1±NI<br>Overall: 84.63±NI<br>Gender:<br>M: NI<br>F: NI	Country<br>Italy<br>Setting:<br>Community: NA<br>Hospital: NA<br>Institution: 31 (100%)<br>Population:<br>Elderly	Experimental: Dog-assisted therapy<br>Frequency: 1 session/week<br>Duration: 30 min<br>Total sessions: 10<br>Format: Group<br>Animal: Dog<br>Control: No treatment	Physical<br>Pain<br>-NRS<br>Psychological<br>Depression<br>-GDS-15<br>Anxiety<br>-GAD-7<br>Affect<br>-PANAS<br>Illness perception<br>-IPQ-R<br>Others: NA	1. Baseline<br>2. Post-test (10 weeks)	High risk
Antonioli et al. (2005) [3]	Sample size<br>Overall: 30<br>EG: 15 (50.0%)<br>CG: 15 (50.0%)<br>Mean (SD) age (years):<br>EG: 41.0±12.5<br>CG: 39.5±10.8<br>Overall: 40.2±11.5<br>Gender:<br>M: 3 (10.0%)<br>F: 27 (90.0%)	Country<br>US and Honduras<br>Setting:<br>Community: 30 (100%)<br>Hospital: NA<br>Institution: NA<br>Population:<br>Depression	Experimental: Animal care program<br>Frequency: 5 sessions/week<br>Duration: 60 min<br>Total sessions: 10<br>Format: Group<br>Animal: Dolphins<br>Control: Outdoor nature program	Physical: NA<br>Psychological:<br>Depression<br>-HDRS<br>-BDI<br>Anxiety<br>-Zung SAS<br>Others: NA	3. Baseline<br>4. Post-test (2 weeks)	Some concerns

Berget et al. (2011) [4]	Sample size<br>Overall: 90<br>EG: 60 (66.7%)<br>CG: 30 (33.3%)<br>Mean (SD) age (years):<br>EG: NI<br>CG: NI<br>Overall: 34.7±10.7<br>Gender:<br>M: 31 (34.4%)<br>F: 59 (65.6%)	Country<br>Norway<br>Setting:<br>Community: 76 (84.4%)<br>Hospital: 14 (15.6%)<br>Institution: NI<br>Population:<br>Psychiatric inpatients and outpatients	Experimental: AAT<br>Frequency: 2 sessions/week<br>Duration: NI<br>Total sessions: 24<br>Format: Individual/Group<br>Animal: Horses and small animals<br>Control: Treatment as usual	Physical: NA<br>Psychological:<br>Anxiety<br>-STAI<br>Depression<br>-BDI<br>Others: NA	5. Baseline<br>6. Post-test (12 weeks)<br>7. Follow-up (6 months post-intervention)	Some concerns
Binfet (2017) [5]	Sample size<br>Overall: 163<br>EG: 84 (51.53%)<br>CG: 79 (48.47%)<br>Mean (SD) age (years):<br>EG: NI<br>CG: NI<br>Overall: 18.85±2.65<br>Gender:<br>M: 36 (22.09%)<br>F: 127 (77.91%)	Country<br>Canada<br>Setting:<br>Community: 163 (100.0%)<br>Hospital: NA<br>Institution: NA<br>Population:<br>Undergraduate students	Experimental: Group-administered canine therapy<br>Frequency: 1 session/week<br>Duration: 20 min<br>Total sessions: 1<br>Format: Group<br>Animal: Dog<br>Control: No treatment	Physical: NA<br>Psychological:<br>Stress<br>-PSS<br>Homesickness<br>-HQ<br>Belonging<br>-SBS<br>Others: NA	8. Baseline<br>9. Post-test (1 week)<br>10. Follow-up (2 weeks after post-test)	High risk
Bono et al. (2015) [6]	Sample size<br>Overall: 24<br>EG: 12 (50.0%)<br>CG: 12 (50.0%)<br>Mean (SD) age (years):<br>EG: 82.1±6.2<br>CG: 78.3±10.3<br>Overall: 80.2±8.54<br>Gender:<br>M: 8 (33.3%)<br>F: 16 (66.7%)	Country<br>Italy<br>Setting:<br>Community: 24 (100.0%)<br>Hospital: NI<br>Institution: NI<br>Population:<br>Minor to mild Alzheimer's disease	Experimental: AAT<br>Frequency: 1 session/bi-weekly<br>Duration: 60 min<br>Total sessions: 32<br>Format: Group<br>Animal: Dog<br>Control: No intervention at home	Physical: NA<br>Psychological:<br>Depression<br>- CSDD<br>Others:<br>ADL<br>- Barthel Index<br>Global cognition<br>- ADAS	11. Baseline<br>12. Post-test (32 weeks)	Some concerns
Chen et al. (2021) [7]	Sample size<br>Overall: 40<br>EG: 20 (50.0%)<br>CG: 20 (50.0%)<br>Mean (SD) age (years):<br>EG: 55.3±11.9	Country<br>Taiwan<br>Setting:<br>Community: NA<br>Hospital: 40 (100%)<br>Institution: NA	Experimental: Animal-assisted therapy<br>Frequency: 1 session/week<br>Duration: 65 min<br>Total sessions: 12	Physical: NA<br>Psychological:<br>Happiness<br>-CHI<br>Depression<br>-DASS-21<br>Anxiety	13. Baseline<br>14. Post-test (12 weeks)	Low risk

	CG: 54.1±13.7<br>Overall: 54.7±12.68<br>Gender:<br>M: 18 (45.5%)<br>F: 22 (55.0%)	Population:<br>Schizophrenia	Format: Group<br>Animal: Dog<br>Control: Treatment as usual	-DASS-21<br>Stress<br>-DASS-21<br>Others:<br>Psychiatric symptoms<br>-PANSS		
Chen et al. (2022) [8]	Sample size<br>Overall: 40<br>EG: 20 (50.0%)<br>CG: 20 (50.0%)<br>Mean (SD) age (years):<br>EG: NI<br>CG: NI<br>Overall: 54.6±NI<br>Gender:<br>M: 18 (45.0%)<br>F: 22 (55.0%)	Country<br>Taiwan<br>Setting:<br>Community: NA<br>Hospital: 40 (100%)<br>Institution: NA<br>Population:<br>Schizophrenia	Experimental: Animal-assisted therapy<br>Freq Treatment as usual: 1 session/week<br>Duration: 65 min<br>Total sessions: 12<br>Format: Group<br>Animal: Dog<br>Control:	Physical:<br>Balance<br>-CST<br>Gait<br>-TUG<br>-5MWT<br>Psychological: NA<br>Others:<br>Communication and interpersonal skills<br>-ACIS<br>Global cognition<br>-MoCA	15. Baseline<br>16. Post-test (12 weeks)	Low risk
Clark et al. (2020) [9]	Sample size<br>Overall: 221<br>EG: 110 (49.8%)<br>CG: 111 (50.2%)<br>Mean (SD) age (years):<br>EG: NI<br>CG: NI<br>Overall: NI<br>Gender:<br>M: 17 (7.7%)<br>F: 204 (92.3%)	Country<br>US<br>Setting:<br>Community: NA<br>Hospital: 221 (100%)<br>Institution: NA<br>Population:<br>Fibromyalgia	Experimental: Animal-assisted activity<br>Frequency: 1session/week<br>Duration: 20 min<br>Total sessions: 1<br>Format: NI<br>Animal: Dog<br>Control: Handler only	Physical:<br>Pain<br>-NRS<br>Physical index<br>-TT<br>-Oxytocin<br>Biomarker<br>-cortisol<br>Fatigue<br>-VAS<br>Psychological:<br>Attitude<br>-PAS<br>Attachment<br>-LPAS<br>Anxiety<br>-VAS<br>Stress<br>-VAS<br>Depression<br>- VAS<br>Happiness<br>- VAS<br>Calm<br>- VAS<br>Well-being<br>- VAS<br>Restlessness<br>- VAS	17. Baseline<br>18. Post-test (1 week)<br>19. Follow-up (2 weeks post-intervention)	Some concerns

				Relaxed<br>- VAS<br>Energetic<br>- VAS<br>Others: NA		
Colombo et al. (2006) [10]	Sample size<br>Overall: 144<br>EG: 48 (33.33%)<br>CG1: 43 (29.86%)<br>CG2: 53 (36.81%)<br>Mean (SD) age (years):<br>EG: 78.4±9.5<br>CG1: 78.8±10.4<br>CG2: 79.5±9.9<br>Overall: 78.8±9.4<br>Gender:<br>M: 47 (32.64%)<br>F: 97 (67.36%)	Country<br>Italy<br>Setting:<br>Community: NA<br>Hospital: NA<br>Institution: 144 (100%)<br>Population: Healthy elderly	Experimental: Group with animals<br>Frequency: 3 sessions/week<br>Duration: 15 min<br>Total sessions: 36<br>Format: NI<br>Animal: Canary<br>Control-I: Routine care<br>Control-II: Plant	Physical: NA<br>Psychological:<br>Anxiety<br>-BSI<br>Others:<br>Quality of life<br>- LEIPAD-PFS<br>Global cognition<br>-MMSE	20. Baseline<br>21. Post-test (3 months)	Some concerns
Fernández-Jorge et al. (2013) [11]	Sample size<br>Overall: 11<br>EG: 6 (54.5%)<br>CG: 5 (45.5%)<br>Mean (SD) age (years):<br>EG: 53.0±12.9<br>CG: 45.8±10.9<br>Overall: 49.73±12.0<br>Gender:<br>M: 6 (54.5%)<br>F: 5 (45.5%)	Country<br>Spain<br>Setting:<br>Community: NA<br>Hospital: NA<br>Institution: 11 (100.0%)<br>Population:<br>Severe and enduring mental disorder	Experimental: AAT<br>Frequency: 2 sessions/week<br>Duration: 60 min<br>Total sessions: 24<br>Format: NI<br>Animal: Dog<br>Control: Social skill, creativity, and cognitive activities	Physical: NA<br>Psychological:<br>Depression<br>-BDI-II<br>Anxiety<br>-STAI<br>-SADS<br>Others:<br>Quality of life<br>-CSCV	22. Baseline<br>23. Post-test (12 weeks)<br>24. Follow-up (12 weeks post-intervention)	Some concerns
Grubbs et al. (2016) [12]	Sample size<br>Overall: 12<br>EG: 7 (58.3%)<br>CG: 5 (41.7%)<br>Mean (SD) age (years):<br>EG: 74.0±5.0<br>CG: 82.0±5.0<br>Overall: 77.3±6.3<br>Gender:<br>M: 3 (25.0%)	Country<br>US<br>Setting:<br>Community: 12 (100.0%)<br>Hospital: NA<br>Institution: NA<br>Population:<br>Older adults	Experimental: Animal-assisted therapy with exercise<br>Frequency: 3 sessions/week<br>Duration: 45 min<br>Total sessions: 18<br>Format: Group<br>Animal: Dog<br>Control: Exercise only group	Physical:<br>Balance<br>-CST<br>-One-leg stand<br>Physical index<br>-BP<br>-HR<br>-BW<br>-BH<br>Flexibility<br>-Chair sit-and-reach<br>- Back scratch test	25. Baseline<br>26. Post-test (6 weeks)	Some concerns

	F: 9 (75.0%)			Gait<br>-8-foot up-and-go test<br>-6-min walk<br>Muscle strength<br>-hand grip<br>Psychological:<br>Depression<br>-GDS-S<br>Others:<br>Quality of life<br>-WHOQOL-BREF		
Jessen et al. (1996) [13]	Sample size<br>Overall: 40<br>EG: 20 (50.0%)<br>CG: 20 (50.0%)<br>Mean (SD) age (years):<br>EG: NI<br>CG: NI<br>Overall: 76.0±NI<br>Gender:<br>M: 13 (32.5%)<br>F: 27 (67.5%)	Country<br>Switzerland<br>Setting:<br>Community: NA<br>Hospital: NA<br>Institution: 40 (100%)<br>Population: Healthy elderly	Experimental: Bird therapy<br>Frequency: NI<br>Duration: 14,400 min (continued for 10 days)<br>Total sessions: NI<br>Format: NI<br>Animal: Bird<br>Control: Treatment as usual	Physical: NA<br>Psychological:<br>Depression<br>-GDS<br>Loneliness<br>-R-UCLALS<br>Others: NA	27. Baseline<br>28. Post-test (10 days)	High risk
Jøranson et al. (2015) [14]	Sample size<br>Overall: 51<br>EG: 27 (50.94%)<br>CG: 26 (49.06%)<br>Mean (SD) age (years):<br>EG: 83.9±7.2<br>CG: 84.1±6.7<br>Overall: 84±NI<br>Gender:<br>M: 18 (33.97%)<br>F: 35 (66.03%)	Country<br>Norway<br>Setting:<br>Community: NA<br>Hospital: NA<br>Institution: 53 (100%)<br>Population: Dementia	Experimental: Paro group<br>Frequency: 2 sessions/week<br>Duration: 30 min<br>Total sessions: 16<br>Format: NI<br>Animal: Paro (Harp seal)<br>Control: Treatment as usual	Physical: NA<br>Psychological:<br>Depression<br>- CSDD<br>Agitation<br>-BARS<br>Others: NA	29. Baseline<br>30. Post-test (2 months)<br>31. Follow-up (3 months after post-test)	Some concerns

Ko et al. (2016) [15]	Sample size<br>Overall: 94<br>EG: 46 (48.9%)<br>CG: 48 (51.1%)<br>Mean (SD) age (years):<br>EG: 71.07±5.51<br>CG: 72.65±5.73<br>Overall: 71.88±5.65<br>Gender:<br>M: 18 (19.1%)<br>F: 76 (80.9%)	Country<br>South Korea<br>Setting:<br>Community: 94 (100.0%)<br>Hospital: NA<br>Institution: NA<br>Population:<br>Healthy elderly	Experimental: Pet insect-assisted therapy<br>Frequency: continued for 8 weeks<br>Duration: NI<br>Total sessions: NI<br>Format: NI<br>Animal: Crickets<br>Control: Healthcare advice	Physical:<br>Sleep<br>-ISI<br>Fatigue<br>-FSS<br>Biomarker<br>-ESR<br>-hsCRP<br>-d-ROMs<br>-BAP<br>Psychological:<br>Anxiety<br>-BAI<br>Depression<br>-GDS-15<br>Stress<br>-BEPSI<br>Others:<br>Quality of life<br>-SF-36<br>Global cognition<br>-MMSE	32. Baseline<br>33. Post-test (8 weeks)	Some concerns
Le Roux & Kemp (2009) [16]	Sample size<br>Overall: 16<br>EG: 8 (50.0%)<br>CG: 8 (50.0%)<br>Mean (SD) age (years):<br>EG: NI<br>CG: NI<br>Overall: NI<br>Gender:<br>M: 8 (50.0%)<br>F: 8 (50.0%)	Country<br>South Africa<br>Setting:<br>Community: NA<br>Hospital: NA<br>Institution: 16 (100.0%)<br>Population:<br>Older adults	Experimental: Animal-assisted activity<br>Frequency: 1 session/week<br>Duration: 30 min<br>Total sessions: 6<br>Format: NI<br>Animal: Dog<br>Control: No intervention	Physical: NA<br>Psychological:<br>Depression<br>-BDI<br>Anxiety<br>-BAI<br>Others: NA	34. Baseline<br>35. Post-test (6 weeks)	Some concerns
Liang et al. (2017) [17]	Sample size<br>Overall: 24<br>EG: 13 (54.17%)<br>CG: 11 (45.83%)<br>Mean (SD) age (years):<br>EG: NI<br>CG: NI<br>Overall: NI<br>Gender:<br>M: NI<br>F: NI	Country<br>New Zealand<br>Setting:<br>Community: NA<br>Hospital: NA<br>Institution: 24 (100%)<br>Population:<br>Dementia	Experimental: Paro group<br>Frequency: 2 sessions/week<br>Duration: 30 min<br>Total sessions: 12<br>Format: NI<br>Animal: Paro (Harp seal)<br>Control: Treatment as usual	Physical:<br>Physical index<br>- BP<br>- HR<br>Psychological:<br>Depression<br>- CSDD<br>Agitation<br>-CMAI-SF<br>Others:<br>Psychiatric symptoms<br>-NPI	36. Baseline<br>37. Post-test (6 weeks)<br>38. Follow-up (6 weeks after post-test)	Some concerns

Mahoney et al. (2024) [18]	Sample size<br>Overall: 21<br>EG: 10 (47.62%)<br>CG:11 (52.38%)<br>Mean (SD) age (years):<br>EG: NI<br>CG: NI<br>Overall: NI<br>Gender:<br>M: 6 (28.57%)<br>F: 15 (71.43%)	Country<br>US<br>Setting:<br>Community: NA<br>Hospital: NA<br>Institution: 21 (100.0%)<br>Population:<br>Cancer pediatric patients with families	Experimental: Animal-assisted interaction<br>Frequency: 1 session/week<br>Duration:15 min<br>Total sessions: 4<br>Format: NI<br>Animal: Dog<br>Control: No treatment	Physical: NA<br>Psychological:<br>Anxiety<br>-STAI<br>Others: NA	39. Baseline<br>40. Post-test (4 weeks)	Some concerns
Majic et al. (2013) [19]	Sample size<br>Overall: 54<br>EG: 27 (50.0%)<br>CG: 27 (50.0%)<br>Mean (SD) age Years):<br>EG: 81.33±10.2<br>CG: 82.07±8.65<br>Overall: 81.7±9.37<br>Gender:<br>M: 16 (29.63%)<br>F: 38 (70.37%)	Country<br>Germany<br>Setting:<br>Community: NA<br>Hospital: NA<br>Institution: 54 (100%)<br>Population:<br>Nursing home residents with dementia	Experimental: AAT<br>Frequency: 1 session/week<br>Duration: 145 min<br>Total sessions: 1<br>Format: NI<br>Animal: Dog<br>Control: No treatment	Physical: NA<br>Psychological:<br>Depression<br>-DMAS<br>Agitation<br>-CMAI<br>Others: NA	41. Baseline<br>42. Post-test (1 week)	Some concerns
Marcus et al. (2013) [20]	Sample size<br>Overall: 133<br>EG: 84 (63.16%)<br>CG:49 (36.84%)<br>Mean (SD) age (years):<br>EG: 48.0±11.5<br>CG: 47.8±14.4<br>Overall: 48.5±NI<br>Gender:<br>M: 4 (.0%)<br>F: 129 (97.0%)	Country<br>US<br>Setting:<br>Community: NA<br>Hospital: NA<br>Institution: 133 (100.0%)<br>Population:<br>Fibromyalgia patients	Experimental: AAT<br>Frequency: 1~2 sessions/week<br>Duration: 15 min<br>Total sessions: 1~2<br>Format: NI<br>Animal: Dog<br>Control: Reading with magazine	Physical:<br>Pain<br>-NRS<br>Psychological:<br>Anxiety<br>-NRS<br>Stress<br>-CSS<br>Others: NA	43. Baseline<br>44. Post-test (15 min)	Some concerns

McCullough et al. (2018) [21]	Sample size<br>Overall: 124<br>EG: 71 (57.26%)<br>CG: 53 (42.74%)<br>Mean (SD) age (years):<br>EG: NI<br>CG: NI<br>Overall: NI<br>Gender:<br>M: NI<br>F: NI	Country<br>US<br>Setting:<br>Community: NA<br>Hospital: 124 (100.0%)<br>Institution: NA<br>Population:<br>Pediatric oncology patients' families	Experimental: Animal-assisted intervention<br>Frequency: 1 session/week<br>Duration: 10~20 min<br>Total sessions: 16<br>Format: NI<br>Animal: Dog<br>Control: No treatments	Physical:<br>Physical index<br>- HR<br>- BP<br>Psychological:<br>Anxiety<br>-STAI<br>Stress<br>-PIP<br>Others: NA	45. Baseline<br>46. Post-test (16 weeks)	Some concerns
Mittly et al. (2024) [22]	Sample size<br>Overall: 116<br>EG: 58 (50.00%)<br>CG: 58 (50.00%)<br>Mean (SD) age (years):<br>EG: NI<br>CG: NI<br>Overall: NI<br>Gender:<br>M: 43 (37.1%)<br>F: 73 (62.9%)	Country<br>Hungary<br>Setting:<br>Community: NA<br>Hospital: NA<br>Institution: 118 (100.0%)<br>Population:<br>Disabilities	Experimental: Dog therapy and physical therapy<br>Frequency: 1 session/week<br>Duration: 120~180 min<br>Total sessions: 3<br>Format: Group<br>Animal: Dog<br>Control: Physical therapy only	Physical:<br>Pain<br>-VAS<br>Psychological:<br>Depression<br>-BDI-9<br>Anxiety<br>-STAI<br>Well-being<br>-WBI<br>Others:<br>Illness Intrusiveness<br>-IIRS	47. Baseline<br>48. Post-test (3 weeks)	Some concerns
Olsen et al. (2016) [23]	Sample size<br>Overall: 51<br>EG: 26 (51.0%)<br>CG: 25 (49.0%)<br>Mean (SD) age (years):<br>EG: 82.9±8.5<br>CG: 84.1±6.7<br>Overall: 83.48±7.62<br>Gender:<br>M: 19 (37.3%)<br>F: 32 (62.7%)	Country<br>Norway<br>Setting:<br>Community: NA<br>Hospital: NA<br>Institution: 51 (100.0%)<br>Population:<br>Dementia	Experimental: Animal-assisted activities<br>Frequency: 2 sessions/week<br>Duration: 30 min<br>Total sessions: 36<br>Format: Group<br>Animal: Dog<br>Control: Treatment as usual	Physical: NA<br>Psychological:<br>Depression<br>- CSDD<br>Agitation<br>-BARS<br>Others:<br>Quality of life<br>-QUALID<br>Global cognition<br>-CDR	49. Baseline<br>50. Post-test (12 weeks)<br>51. Follow-up (3 months post-intervention)	Some concerns

Pedersen et al. (2011) [24]	Sample size<br>Overall: 29<br>EG: 16 (55.2%)<br>CG: 13 (44.8%)<br>Mean (SD) age (years):<br>EG :40.5±10.7<br>CG: 34.0±6.6<br>Overall: 37.58±9.53<br>Gender:<br>M: 6 (20.7%)<br>F: 23 (79.3%)	Country<br>Norway<br>Setting:<br>Community: 29 (100%)<br>Hospital: NA<br>Institution: NA<br>Population:<br>Depression	Experimental: Farm animal-assisted intervention<br>Frequency: 2 sessions/week<br>Duration: 90-180 min<br>Total sessions: 24<br>Format: NI<br>Animal: Farm animals<br>Control: Waiting list control	Physical: NA<br>Psychological:<br>Depression<br>-BDI<br>Anxiety<br>-STAI-subscale<br>Self-efficacy<br>-GSE<br>Others: NA	52. Baseline<br>53. Mid-test (4 weeks)<br>54. Mid-test (8 weeks)<br>55. Post-test (12 weeks)<br>56. Follow-up (3 months post-intervention)	Some concerns
Petersen et al. (2017) [25]	Sample size<br>Overall: 61<br>EG: 35 (57.4%)<br>CG: 26 (42.6%)<br>Mean (SD) age (years):<br>EG: 83.5±5.8<br>CG: 83.3±6.0<br>Overall: 83.41±5.83<br>Gender:<br>M: 14 (23.0%)<br>F: 47 (77.0%)	Country<br>US<br>Setting:<br>Community: NA<br>Hospital: NA<br>Institution: 61 (100.0%)<br>Population:<br>Dementia	Experimental: Robotic pet assisted<br>Frequency: 3 sessions/week<br>Duration: 20 min<br>Total sessions: 36<br>Format: Group<br>Animal: Seal<br>Control: Treatment as usual	Physical:<br>Physical index<br>-HR<br>-Pulse oximetry<br>Psychological:<br>Depression<br>-CSDD<br>Anxiety<br>-RAID<br>Stress<br>-GSR<br>Others: NA	57. Baseline<br>58. Post-test (12 weeks)	Some concerns
Riddick et al. (1985) [26]	Sample size<br>Overall: 22<br>EG: 7 (31.82%)<br>CG1: 8 (36.36%)<br>CG2: 7 (31.82%)<br>Mean (SD) age (years):<br>EG: NI<br>CG1: NI<br>CG2: NI<br>Overall: NI<br>Gender:<br>M: NI<br>F: NI	Country<br>US<br>Setting:<br>Community: 22 (100.0%)<br>Hospital: NA<br>Institution: NA<br>Population: Elderly	Experimental: Aquarium group<br>Frequency: 1 session/2 weeks<br>Duration: 10-15 min<br>Total sessions: 12<br>Format: Group<br>Animal: Fish<br>Control-I: Routine care (no visitor and tank)<br>Control-II: Visitor only (tank maintenance visits)	Physical:<br>Physical index<br>-BP<br>Psychological:<br>Anxiety<br>-STAI<br>Happiness<br>-MUNSH<br>Loneliness<br>-UCLALS<br>Satisfaction<br>-LSS<br>Others: NA	59. Baseline<br>60. Post-test (6 months)	High risk

Robinson et al. (2013) [27]	Sample size<br>Overall: 40<br>EG: 20 (50.0%)<br>CG: 20 (50.0%)<br>Mean (SD) age (years):<br>EG: NI<br>CG: NI<br>Overall: NI<br>Gender:<br>M: 13 (33.0%)<br>F: 27 (67.0%)	Country<br>New Zealand<br>Setting:<br>Community: NA<br>Hospital: 22(55.0%)<br>Institution: 18(45.0%)<br>Population: Cognitive impairment	Experimental: Paro robot<br>Frequency: 2 sessions/week<br>Duration: 60 min<br>Total sessions: 24<br>Format: NI<br>Animal: Robot dog<br>Control: Bus trips	Physical: NA<br>Psychological:<br>Depression<br>-GDS<br>Loneliness<br>-UCLALS<br>Others:<br>Quality of life<br>-Qol-AD	61. Baseline<br>62. Post-test (12 weeks)	Some concerns
Rodrigo-Claverol et al. (2019) [28]	Sample size<br>Overall: 52<br>EG: 30 (57.69%)<br>CG: 22 (42.31%)<br>Mean (SD) age (years):<br>EG: 74.2±7.08<br>CG: 77.5±7.3<br>Overall: 77.5±7.3<br>Gender:<br>M: 5 (9.62%)<br>F: 47 (90.38%)	Country<br>Spain<br>Setting:<br>Community: NA<br>Hospital: NA<br>Institution: 52 (100.0%)<br>Population:<br>Chronic joint pain	Experimental: Animal-assisted intervention with exercise<br>Frequency: 12 sessions/week<br>Duration: 60 min<br>Total sessions: 12<br>Format: NI<br>Animal: Dog<br>Control: Exercise only	Physical:<br>Pain<br>-VAS<br>Physical disability<br>-WOMAC<br>Psychological:<br>Depression<br>-GDS<br>Others:<br>Perceived health<br>-HAQ<br>Quality of life<br>-EQ-5D	63. Baseline<br>64. Post-test (1 week)	Some concerns
Thirion et al. (2023) [29]	Sample size<br>Overall: 42<br>EG: 22 (52.4%)<br>CG: 20 (47.6%)<br>Mean (SD) age (years):<br>EG: 81.4±6.02<br>CG: 82.5±4.91<br>Overall: 81.92±5.48<br>Gender:<br>M: 18 (42.9%)<br>F: 24 (57.1%)	Country<br>France<br>Setting:<br>Community: NA<br>Hospital: NA<br>Institution:42 (100.0%)<br>Population:<br>Alzheimer's disease	Experimental: AAT<br>Frequency: 1 session/week<br>Duration: 60 min<br>Total sessions: 8<br>Format: Group<br>Animal: Dog<br>Control: Stimulation of cognition	Physical: NA<br>Psychological:<br>Well-being<br>-EVIBE<br>Depression<br>-GDS<br>Anxiety<br>-STAI<br>Others:<br>Psychiatric symptoms<br>-NPI<br>Global cognition<br>-ADAS	65. Baseline<br>66. Post-test (8 weeks)<br>67. Follow-up (4 weeks post-intervention)	Some concerns

Thompkins et al. (2019) [30]	Sample size<br>Overall: 31<br>EG: 16 (51.61%)<br>CG: 15 (48.39%)<br>Mean (SD) age (years):<br>EG: 35.43±16.08<br>CG: 41.75±16.12<br>Overall: 38.59±NI<br>Gender:<br>M: 22 (70.97%)<br>F: 9 (29.03%)	Country<br>US<br>Setting:<br>Community: NA<br>Hospital: NA<br>Institution: 31 (100.0%)<br>Population:<br>Spinal cord injury	Experimental: AAT<br>Frequency: 4 sessions/week<br>Duration: 30 min<br>Total sessions: 4<br>Format: NI<br>Animal: Dog<br>Control: Occupational therapy	Physical:<br>Biomarker<br>-cortisol<br>Pain<br>-NRS<br>-BPI<br>Psychological:<br>Affect<br>-PANAS<br>Others: NA	68. Baseline<br>69. Post-test (1 week)	High risk
Travers et al. (2005) [31]	Sample size<br>Overall: 55<br>EG: 27 (49.10%)<br>CG: 28 (50.90%)<br>Mean (SD) age (years):<br>EG: 84.9±6.1<br>CG: 85.1±6.6<br>Overall: 85.0±NI<br>Gender:<br>M: 12 (21.82%)<br>F: 43 (78.18%)	Country<br>Australia<br>Setting:<br>Community: NA<br>Hospital: NA<br>Institution: 55 (100%)<br>Population:<br>Dementia residents	Experimental: Dog-assisted therapy<br>Frequency: 2 sessions/week<br>Duration: 40–50 min<br>Total sessions: 22<br>Format: NI<br>Animal: Dog<br>Control: Human therapist only	Physical: NA<br>Psychological:<br>Depression<br>-GDS-S<br>Others:<br>Psychiatric symptoms<br>-MOSES<br>Quality of life<br>-SF-36<br>-QoL-AD<br>Concentration<br>-VAS	70. Baseline<br>71. Post-test (11 weeks)	Some concerns
Vegue Parra et al. (2021) [32]	Sample size<br>Overall: 334<br>EG: 171 (51.19%)<br>CG: 163 (48.81%)<br>Mean (SD) age (years):<br>EG: 53.0±12.9<br>CG: 45.8±10.9<br>Overall: NI<br>Gender:<br>M: 75 (22.46%)<br>F: 259 (77.54%)	Country<br>Spain<br>Setting:<br>Community: NA<br>Hospital: NA<br>Institution: 3711[??] (100.0%)<br>Population:<br>Neurocognitive disorder	Experimental: Dog-assisted therapy<br>Frequency: 1 session/week<br>Duration: 45 min<br>Total sessions: 32<br>Format: Group<br>Animal: Dog<br>Control: Treatment as usual	Physical: NA<br>Psychological:<br>Depression<br>- CSDD<br>Others:<br>Psychiatric symptoms<br>-NPI<br>ADL<br>-Modified-Barthel index<br>Global cognition<br>-MMSE	72. Baseline<br>73. Mid-test (12 weeks)<br>74. Post-test (32 weeks)	Some concerns

Vignolo et al. (2024) [33]	Sample size<br>Overall: 60<br>EG: 30 (50.0%)<br>CG: 30 (50.0%)<br>Mean (SD) age (years):<br>EG: 65.55±9.83<br>CG: 69.60±9.25<br>Overall: 67.33±NI<br>Gender:<br>M: 38 (63.0%)<br>F: 22 (37.0%)	Country<br>Italy<br>Setting:<br>Community: NA<br>Hospital: NA<br>Institution: 60 (100.0%)<br>Population:<br>Amyotrophic lateral sclerosis	Experimental: Dog-assisted physiotherapy<br>Frequency: 2 sessions/week<br>Duration: 60 min<br>Total sessions: 2<br>Format: NI<br>Animal: Dog<br>Control: Regular balance exercise	Physical:<br>Gait<br>-TUG<br>-TSMWT<br>-TTMWT<br>Balance<br>-SPPB<br>Psychological:<br>Anxiety<br>-HADS<br>Depression<br>-HADS<br>Others: NA	75. Baseline<br>76. Post-test (2 weeks)	Some concerns
Wijker et al. (2020) [34]	Sample size<br>Overall: 53<br>EG: 27 (50.9%)<br>CG: 26 (49.1%)<br>Mean (SD) age (years):<br>EG: NI<br>CG: NI<br>Overall: NI<br>Gender:<br>M: 29 (54.7%)<br>F: 24 (45.3%)	Country<br>Netherlands<br>Setting:<br>Community: 53 (100.0%)<br>Hospital: NA<br>Institution: NA<br>Population:<br>Autism spectrum disorder	Experimental: Animal-assisted therapy<br>Frequency: 10 sessions weekly<br>Duration: 60 min<br>Total sessions: 10<br>Format: Individual<br>Animal: Dog<br>Control: Waiting list control	Physical: NA<br>Psychological:<br>Stress<br>-PSS<br>Self-esteem<br>-RSES<br>Others:<br>Psychological and physical symptoms<br>-SCL-90R<br>Social Responsiveness<br>-SRS-A	77. Baseline<br>78. Post-test (10 sessions weekly)<br>79. Follow-up (10 weeks)	Some concerns
Wijker et al. (2021) [35]	Sample size<br>Overall: 53<br>EG: 27 (50.9%)<br>CG: 26 (49.1%)<br>Mean (SD) age (years):<br>EG:ˇ38.0±12.49<br>CG: 39.96±10.34<br>Overall: 38.96±11.4<br>Gender:<br>M: 29 (54.7%)<br>F: 24 (`45.3%))	Country<br>Netherlands<br>Setting:<br>Community: 53 (100.0%)<br>Hospital: NA<br>Institution: NA<br>Population:<br>Autism spectrum disorder	Experimental: Animal-assisted therapy<br>Frequency: 10 sessions weekly<br>Duration: 60 min<br>Total sessions: 10<br>Format: Individual<br>Animal: Dog<br>Control: Waiting list control	Physical: NA<br>Psychological:<br>Stress<br>-Cortisol<br>-Alpha-amylase<br>-ECG<br>-ICG<br>-HRV<br>Others: NA	80. Baseline<br>81. Post-test (10 weeks)<br>82. Follow-up (10 weeks)	Some concerns

Abbreviations: EG, experimental group; CG, control group; M, male; F, female; NI, no information; NA, not available; NR, not reported; RoB, Risk of Bias; ADLs, activities of daily living; 5MWT, 5-Meter Walk Test; ACIS, Assessment of Communication and Interaction Skills; ADAS, Alzheimer Disease Assessment Scale; BAI, Beck Anxiety Inventory; BAP, biological antioxidant potential; BARS, Brief Agitation Rating Scale; BDI, Beck Depression Inventory; BEPSI, Brief Encounter Psychosocial Instrument; BH, body height; BP, blood pressure; BPI, Brief Pain Inventory; BSI, Brief Symptom Inventory; BW, body weight; CDR, Clinical Dementia Rating; CDRISC, Connor-Davidson Resilience Scale; CHI, Chinese Happiness Inventory; CMAI, Cohen-Mansfield Agitation Inventory; CMAI-SF, Cohen-Mansfield Agitation Inventory–Short Form; CSCV, Seville Quality of Life Questionnaire; CSDD, Cornell Scale for Depression in Dementia; CSS, Current Symptom Severity; CST, chair stand test; DASS-21, Depression, Anxiety Stress Scales-21; DMAS, Dementia Mood Assessment Scale; d-ROMs, derivatives of reactive oxygen metabolites; ECG, electrocardiogram; EQ-5D, EuroQol-5 Dimension; ESR, erythrocyte sedimentation rate; EVIBE, Échelle d'évaluation Instantanée du Bien-Être; FSS, Fatigue Severity Scale; GAD-7, Generalized Anxiety Disorder 7; GDS-15, 15-Item Geriatric Depression Scale; GDS-S, Geriatric Depression Scale-Short Form; GSE, Generalized Self-Efficacy Scale; GSR, galvanic skin response; HADS, Hospital Anxiety and Depression Scale; HAQ, Health Assessment Questionnaire; HDRS, Hamilton Depression Rating Scale; HQ, Homesickness Questionnaire; HR, heart rate; HRV, heart rate variability; hsCRP, high-sensitivity C-reactive protein; ICG, impedance cardiogram; IIRS, Illness Intrusiveness Rating Scale; IPQ-R, Illness Perception Questionnaire-Revised; ISI, Insomnia Severity Index; LEIPAD-PFS, LEIPAD II-Short Version with Physical Functioning; LPAS, Lexington Pet Attachment Scale; LSS, Leisure Satisfaction Scale; MMSE, Mini-Mental State

Examination; MoCA, Montreal Cognitive Assessment; MOSES, Multidimensional Observation Scale for Elderly Subjects; MOS-SSS, Medical Outcomes Study-Social Support; MUNSH, Memorial University of Newfoundland Scale of Happiness; NPI: Neuropsychiatric Inventory; NRS: Numeric Rating Scale; PANAS: Positive and Negative Affect Schedule; PANSS: Positive and Negative Syndrome Scale; PAS: Pet Attitude Scale; PCL-C: PTSD Checklist Civilian Version; PHQ-15, Patient Health Questionnaire-15; PHQ-9, Patient Health Questionnaire-9; PIP, Pediatric Inventory for Parents; PIRS, Pittsburgh Insomnia Rating Scale; POMS, Profile of Moods States; PSS, Perceived Stress Scale; QOL-AD, Quality of Life in Alzheimer's Disease Scale; QUALID, Quality of Life in Late-Stage Dementia; RAID, Rating for Anxiety in Dementia; RSES, Rosenberg Self-Esteem Scale; R-UCLALS, Revised UCLA Loneliness Scale; SADS, Social Anxiety and Distress Scale; SBS, Sense of Belonging in School; SCL-90-R, Symptom Checklist-90-Revised; SF-36, 36-Item Short-Form Health Survey; SPPB, Short Physical Performance Battery; SRS-A, Social Responsiveness Scale for Adults; STAI, Spielberger State Anxiety Inventory; TSMWT, Test Six Minutes Walk Test; TT, tympanic temperature; TTMWT, Ten Meters Walking Test; TUG, Timed Up-and-Go; UCLALS, UCLA Loneliness Scale; VAS, visual analogue scale; WBI, WHO Well-Being Index; WHOQOL-BREF, World Health Organization Quality of Life Brief Version; WOMAC, Western Ontario and McMaster Universities Arthritis Index; Zung SAS, Zung Self-Rating Anxiety Scale.

Table S3. Studies quality assessment: Randomised controlled study design

Author (Year)	Randomization process	Deviations from intended interventions	Missing outcome data	Measurement of the outcome	Selection of the reported result	Overall bias
Abraham et al. (2021)	?	L	L	L	L	?
Ambrosi et al. (2019)	H	H	L	L	L	H
Antonioli et al. (2005)	L	?	L	L	L	?
Berget et al. (2011)	?	L	L	L	?	?
Binfet (2017)	?	H	L	H	L	H
Bono et al. (2015)	?	?	L	L	?	?
Chen et al. (2021)	L	L	L	L	L	L
Chen et al. (2022)	L	L	L	L	L	L
Clark et al. (2020)	?	L	L	L	L	?
Colombo et al. (2006)	L	L	L	?	L	?
Fernández-Jorge et al. (2013)	?	?	L	L	L	?
Grubbs et al. (2016)	?	?	L	L	?	?
Jessen et al. (1996)	?	H	L	H	L	H
Jøranson et al. (2015)	?	L	L	L	?	?
Ko et al. (2016)	?	L	L	L	L	?
Le Roux & Kemp. (2009)	?	?	L	L	?	?
Liang et al. (2017)	?	?	?	L	L	?
Mahoney et al., (2024)	L	L	L	?	L	?
Majic et al. (2013)	?	?	L	?	L	?
Marcus et al., (2013)	?	?	L	?	L	?
McCullough et al. (2018)	?	?	L	?	L	?

Author (Year)	Randomization process	Deviations from intended interventions	Missing outcome data	Measurement of the outcome	Selection of the reported result	Overall bias
Mittly et al., (2024)	?	?	L	?	L	?
Olsen et al. (2016)	?	?	L	L	?	?
Pedersen et al. (2011)	?	?	L	L	L	?
Petersen et al. (2017)	?	L	L	L	L	?
Riddick et al. (1985)	?	H	L	?	L	H
Robinson et al. (2013)	?	?	L	L	L	?
Rodrigo-Claverol et al. (2019)	?	L	?	?	L	?
Thirion et al. (2023)	?	L	L	L	L	?
Thompkins et al. (2019)	?	?	H	L	L	H
Travers et al. (2005)	?	L	L	L	L	?
Vegue Parra et al. (2021)	?	?	L	L	?	?
Vignolo et al. (2024)	?	?	L	L	L	?
Wijker et al. (2020)	?	L	L	L	?	?
Wijker et al. (2021)	?	L	L	L	?	?

Note: A quality assessment based on the Cochrane Risk of Bias Tool RoB 2.0; Abbreviation: L=Low risk; ?=Some concerns; H=High risk.

Table S4. Moderator analysis of meta-regression for depression, anxiety, and stress in general adults.

Variable	Depression			Anxiety			Stress		
	k	Coefficient	*p*-value	k	Coefficient	*p*-value	k	Coefficient	*p*-value
Mean Age	19	-0.0018	0.7425	13	-0.0027	0.8841	6	0.0003	0.9937
Female (%)	23	0.0016	0.7085	16	-0.0243	0.0749	8	-0.0388	0.0860
Mean total sessions	23	0.0013	0.8430	16	0.0041	0.6390	8	-0.0147	0.2724

Supplementary File 1. List of studies included in the meta-analysis

1. Abraham, P. A., J. B. Kazman, J. A. Bonner, M. D. Olmert, R. A. Yount, and P. A. Deuster. 2021. "Effects of Training Service Dogs on Service Members with PTSD: A Pilot-Feasibility Randomized Study with Mixed Methods." *Mil Psychol* 34, no. 2: 187–196. https://doi.org/10.1080/08995605.2021.1984126

2. Ambrosi, C., C. Zaiontz, G. Peragine, S. Sarchi, and F. Bona. 2019. "Randomized Controlled Study on the Effectiveness of Animal-Assisted Therapy on Depression, Anxiety, and Illness Perception in Institutionalized Elderly." *Psychogeriatrics* 19, no. 1: 55–64. https://doi.org/10.1111/psyg.12367

3. Antonioli, C., and M. A. Reveley. 2005. "Randomised Controlled Trial of Animal Facilitated Therapy with Dolphins in the Treatment of Depression." *BMJ* 331, no. 7527: 1231. https://doi.org/10.1136/bmj.331.7527.1231

4. Berget, B., Ø. Ekeberg, I. Pedersen, and B. O. Braastad. 2011. "Animal-Assisted Therapy with Farm Animals for Persons with Psychiatric Disorders: Effects on Anxiety and Depression, a Randomized Controlled Trial." *Occup Ther Ment Health* 27, no. 1: 50–64.

5. Binfet, J. T. 2017. "The Effects of Group-Administered Canine Therapy on University Students' Wellbeing: A Randomized Controlled Trial." *Anthrozoös* 30, no. 3: 397–414.

6. Bono, A. V., C. Benvenuti, M. Buzzi, R. Ciatti, V. Chiarelli, P. Chiambretto, and E. Valena. 2015. "Effects of Animal-Assisted Therapy (AAT) Carried Out with Dogs on the Evolution of Mild Cognitive Impairment." *G Gerontol* 63, no. 1: 32–36.

7. Chen, T. T., T. L. Hsieh, M. L. Chen, W. T. Tseng, C. F. Hung, and C. R. Chen. 2021. "Animal-Assisted Therapy in Middle-Aged and Older Patients with Schizophrenia: A Randomized Controlled Trial." *Front Psychiatry* 12: 713623. https://doi.org/10.3389/fpsyt.2021.713623

8. Chen, C. R., C. F. Hung, Y. W. Lee, W. T. Tseng, M. L. Chen, and T. T. Chen. 2022. "Functional Outcomes in a Randomized Controlled Trial of Animal-Assisted Therapy on Middle-Aged and Older Adults with Schizophrenia." *Int J Environ Res Public Health* 19, no. 10: 6270. https://doi.org/10.3390/ijerph19106270

9. Clark, S., F. Martin, R. T. S. McGowan, J. Smidt, R. Anderson, L. Wang, T. Turpin, N. Langenfeld-McCoy, B. Bauer, and A. B. Mohabbat. 2020. "The Impact of a 20-Minute Animal-Assisted Activity Session on the Physiological and Emotional States in Patients with Fibromyalgia." *Mayo Clin Proc* 95, no. 11: 2442–2461. https://doi.org/10.1016/j.mayocp.2020.04.037

10. Colombo, G., M. D. Buono, K. Smania, R. Raviola, and D. De Leo. 2006. "Pet Therapy and Institutionalized Elderly: A Study on 144 Cognitively Unimpaired Subjects." *Arch Gerontol Geriatr* 42, no. 2: 207–216. https://doi.org/10.1016/j.archger.2005.06.011

11. Fernández-Jorge, M. T., M. I. Roldán-Gacimartín, M. G. de Gómez-Alfageme, M. L. Vargas, and G. Lahera-Corteza. 2013. "Aplicabilidad y Efectividad de la Terapia Asistida con Animales en Personas con Trastorno Mental Grave y Duradero: Un Ensayo Piloto Aleatorizado." *Rehabilitación Psicosocial* 10, no. 1: 18–24.

12. Grubbs, B., A. Artese, K. Schmitt, E. Cormier, and L. Panton. 2016. "A Pilot Study to Assess the Feasibility of Group Exercise and Animal-Assisted

Therapy in Older Adults." *J Aging Phys Act* 24, no. 2: 322–331. https://doi.org/10.1123/japa.2015-0107
13. Jessen, J., F. Cardiello, and M. M. Baun. 1996. "Avian Companionship in Alleviation of Depression, Loneliness, and Low Morale of Older Adults in Skilled Rehabilitation Units." *Psychol Rep* 78, no. 1: 339–348. https://doi.org/10.2466/pr0.1996.78.1.339
14. Jøranson, N., I. Pedersen, A. M. Rokstad, and C. Ihlebaek. 2015. "Change in Quality of Life in Older People with Dementia Participating in Paro-Activity: A Cluster-Randomized Controlled Trial." *J Adv Nurs* 72, no. 12: 3020–3033. https://doi.org/10.1111/jan.13076
15. Ko, H. J., C. H. Youn, S. H. Kim, and S. Y. Kim. 2016. "Effect of Pet Insects on the Psychological Health of Community-Dwelling Elderly People: A Single-Blinded, Randomized, Controlled Trial." *Gerontology* 62, no. 2: 200–209. https://doi.org/10.1159/000439129
16. Le Roux, M. C., and R. Kemp. 2009. "Effect of a Companion Dog on Depression and Anxiety Levels of Elderly Residents in a Long-Term Care Facility." *Psychogeriatrics* 9, no. 1: 23–26.
17. Liang, A., I. Piroth, H. Robinson, B. MacDonald, M. Fisher, U. M. Nater, N. Skoluda, and E. Broadbent. 2017. "A Pilot Randomized Trial of a Companion Robot for People with Dementia Living in the Community." *J Am Med Dir Assoc* 18, no. 10: 871–878. https://doi.org/10.1016/j.jamda.2017.05.019
18. Mahoney, A. B., T. F. Akard, B. A. Cowfer, M. S. Dietrich, J. L. Newton, and M. J. Gilmer. 2024. "Impact of Animal-Assisted Interaction on Anxiety in Children with Advanced Cancer and Their Caregivers." *J Palliat Med* 27, no. 1: 75–82. https://doi.org/10.1089/jpm.2023.0091
19. Majić, T., H. Gutzmann, A. Heinz, U. E. Lang, and M. A. Rapp. 2013. "Animal-Assisted Therapy and Agitation and Depression in Nursing Home Residents with Dementia: A Matched Case-Control Trial." *Am J Geriatr Psychiatry* 21, no. 11: 1052–1059. https://doi.org/10.1016/j.jagp.2013.03.004
20. Marcus, D. A., C. D. Bernstein, J. M. Constantin, F. A. Kunkel, P. Breuer, and R. B. Hanlon. 2013. "Impact of Animal-Assisted Therapy for Outpatients with Fibromyalgia." *Pain Med* 14, no. 1: 43–51. https://doi.org/10.1111/j.1526-4637.2012.01522.x
21. McCullough, A., A. Ruehrdanz, M. A. Jenkins, M. J. Gilmer, J. Olson, A. Pawar, L. Holley, S. Sierra-Rivera, D. E. Linder, D. Pichette, N. J. Grossman, C. Hellman, N. A. Guérin, and M. E. O'Haire. 2018. "Measuring the Effects of an Animal-Assisted Intervention for Pediatric Oncology Patients and Their Parents: A Multisite Randomized Controlled Trial." *J Pediatr Oncol Nurs* 35, no. 3: 159–177. https://doi.org/10.1177/1043454217748586
22. Mittly, V., V. Fáy, N. Dankovics, V. Pál, and G. Purebl. 2024. "The Role of Dog Therapy in Clinical Recovery and Improving Quality of Life: A Randomized, Controlled Trial." *BMC Complement Med Ther* 24, no. 1: 229. https://doi.org/10.1186/s12906-024-04538-7
23. Olsen, C., I. Pedersen, A. Bergland, M. J. Enders-Slegers, G. Patil, and C. Ihlebaek. 2016. "Effect of Animal-Assisted Interventions on Depression, Agitation and Quality of Life in Nursing Home Residents Suffering from Cognitive Impairment or Dementia: A Cluster Randomized Controlled Trial." *Int J Geriatr Psychiatry* 31, no. 12: 1312–1321. https://doi.org/10.1002/gps.4436
24. Pedersen, I., T. Nordaunet, E. W. Martinsen, B. Berget, and B. O. Braastad. 2011. "Farm Animal-Assisted Intervention: Relationship Between Work and Contact with Farm Animals and Change in Depression, Anxiety, and Self-Efficacy Among Persons with Clinical Depression." *Issues Ment Health Nurs* 32, no.

8: 493–500. https://doi.org/10.3109/01612840.2011.566982
25. Petersen, S., S. Houston, H. Qin, C. Tague, and J. Studley. 2017. "The Utilization of Robotic Pets in Dementia Care." *J Alzheimers Dis* 55, no. 2: 569–574. https://doi.org/10.3233/JAD-160703
26. Riddick, C. C. 1985. "Health, Aquariums, and the Non-Institutionalized Elderly." *Marriage Fam Rev* 8, no. 3–4: 163–173.
27. Robinson, H., B. Macdonald, N. Kerse, and E. Broadbent. 2013. "The Psychosocial Effects of a Companion Robot: A Randomized Controlled Trial." *J Am Med Dir Assoc* 14, no. 9: 661–667. https://doi.org/10.1016/j.jamda.2013.02.007
28. Rodrigo-Claverol, M., C. Casanova-Gonzalvo, B. Malla-Clua, E. Rodrigo-Claverol, J. Jové-Naval, and M. Ortega-Bravo. 2019. "Animal-Assisted Intervention Improves Pain Perception in Polymedicated Geriatric Patients with Chronic Joint Pain: A Clinical Trial." *Int J Environ Res Public Health* 16, no. 16: 2843. https://doi.org/10.3390/ijerph16162843
29. Thirion, E., S. Rouissi, V. Dauphinot, A. Garnier-Crussard, M. H. Coste, and P. Krolak-Salmon. 2023. "Impact de la Thérapie Assistée par l'Animal sur le Bien-Être chez des Patients Présentant une Maladie d'Alzheimer (Étude ELIAUT)." *Gériatr Psychol Neuropsychiatr Vieil* 21, no. 4.
30. Thompkins, A. M., S. J. Adkins, M. Leopard, C. Spencer, D. Bentley, L. Bolden, C. H. Jagielski, E. Richardson, A. M. Goodman, and D. C. Schwebel. 2019. "Dogs as an Adjunct to Therapy: Effects of Animal-Assisted Therapy on Rehabilitation Following Spinal Cord Injury." *Anthrozoös* 32, no. 5: 679–690. https://doi.org/10.1080/08927936.2019.1645513
31. Travers, C., J. Perkins, J. Rand, H. Bartlett, and J. Morton. 2005. "An Evaluation of Dog-Assisted Therapy for Residents of Aged Care Facilities with Dementia." *Anthrozoös* 26, no. 2: 213–225.
32. Vegue Parra, E., J. M. Hernández Garre, and P. Echevarría Pérez. 2021. "Benefits of Dog-Assisted Therapy in Patients with Dementia Residing in Aged Care Centers in Spain." *Int J Environ Res Public Health* 18, no. 4: 1471. https://doi.org/10.3390/ijerph18041471
33. Vignolo, M., R. Zuccarino, R. Truffelli, C. Gemelli, E. Giove, P. M. Ferraro, D. Manunza, C. Trinchero, I. Cipollina, M. Lungu, A. Lizio, G. Gragnano, C. Cabona, M. Pardini, C. Caponnetto, and F. Rao. 2024. "Dog-Assisted Physiotherapy in Amyotrophic Lateral Sclerosis: A Randomized Controlled Pilot Study." *Eur J Phys Rehabil Med* 60, no. 3: 470–476. https://doi.org/10.23736/S1973-9087.24.08343-6
34. Wijker, C., R. Leontjevas, A. Spek, and M. J. Enders-Slegers. 2020. "Effects of Dog-Assisted Therapy for Adults with Autism Spectrum Disorder: An Exploratory Randomized Controlled Trial." *J Autism Dev Disord* 50, no. 6: 2153–2163. https://doi.org/10.1007/s10803-019-03971-9
35. Wijker, C., N. Kupper, R. Leontjevas, A. Spek, and M. J. Enders-Slegers. 2021. "The Effects of Animal-Assisted Therapy on Autonomic and Endocrine Activity in Adults with Autism Spectrum Disorder: A Randomized Controlled Trial." *Gen Hosp Psychiatry* 72: 36–44. https://doi.org/10.1016/j.genhosppsych.2021.05.003

Figure S1. Funnel plot of depression and anxiety

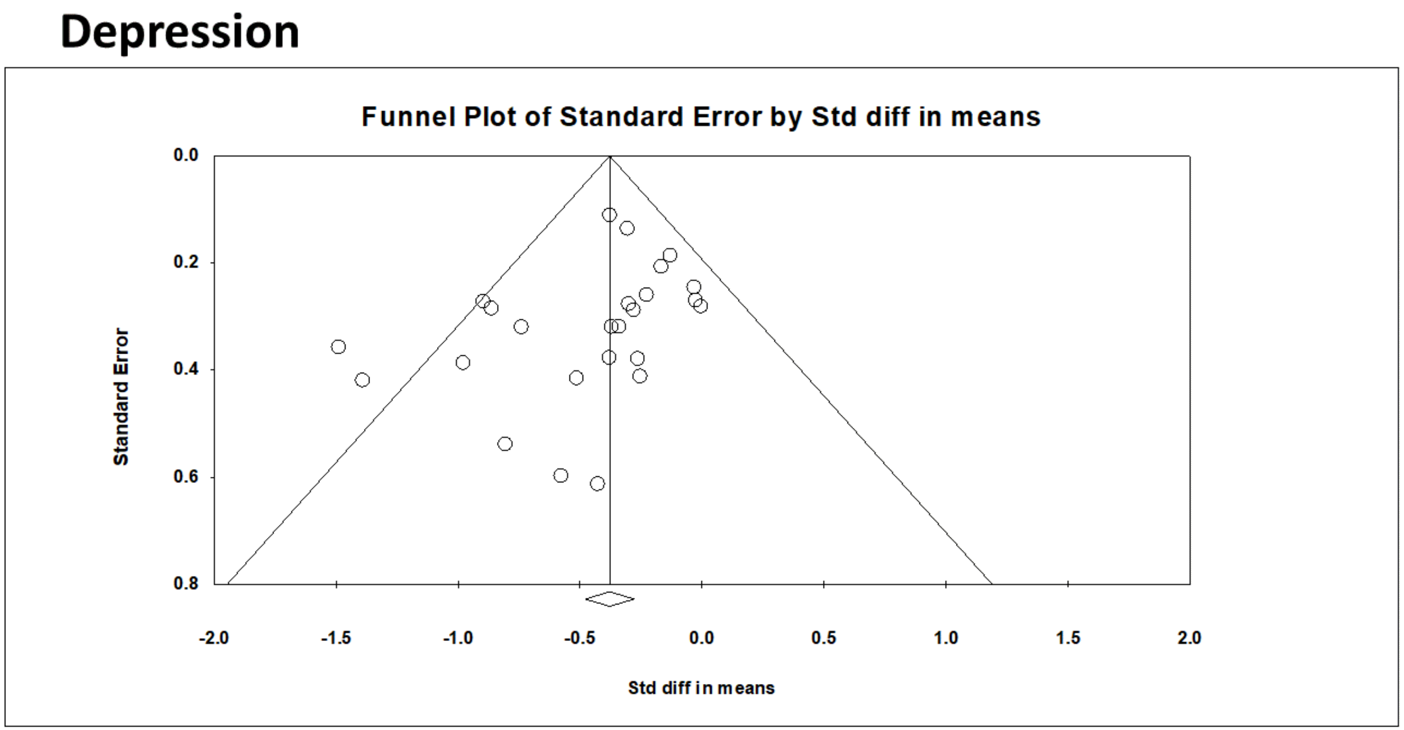

Anxiety

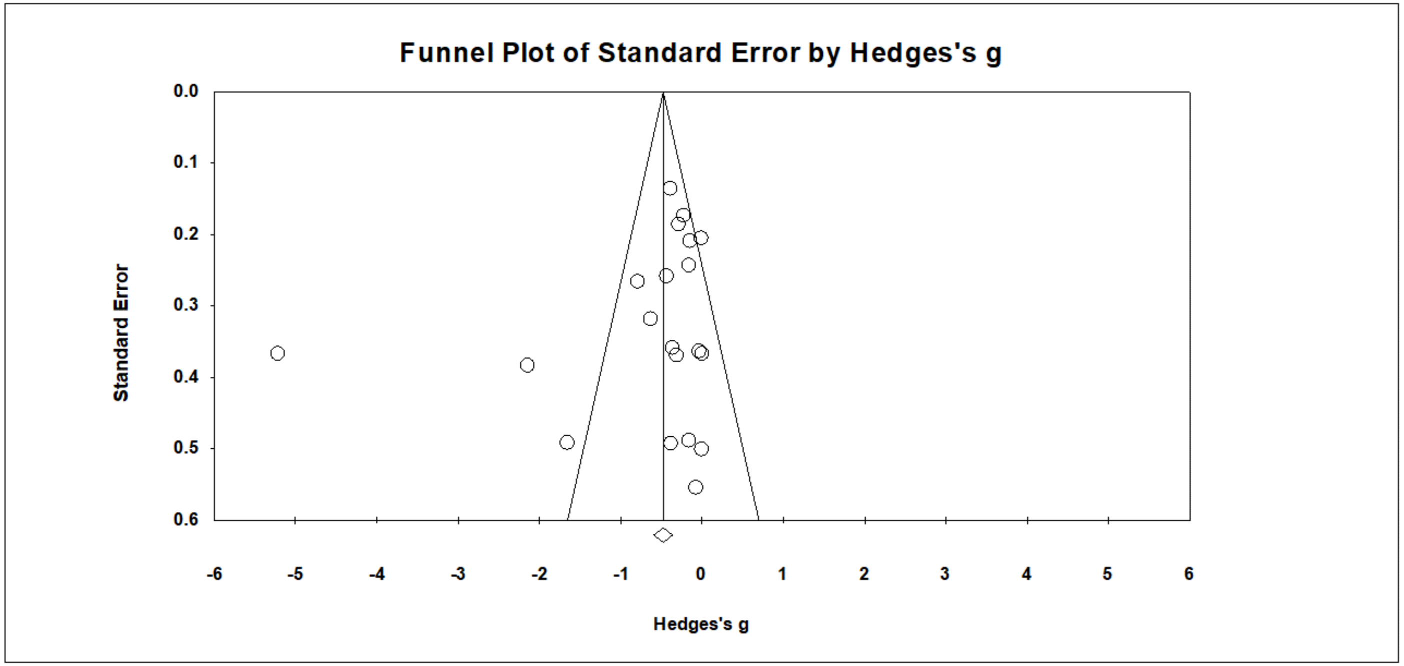
Funnel Plot of Standard Error by Hedges's g
Standard Error
0.0
0.1
0.2
0.3
0.4
0.5
0.6
-6
-5
-4
-3
-2
-1
0
1
2
3
4
5
6
Hedges's g
